# Shared and distinct functional networks for empathy and pain processing: a systematic review and meta-analysis of fMRI studies

**DOI:** 10.1093/scan/nsaa090

**Published:** 2020-06-29

**Authors:** Nicholas Fallon, Carl Roberts, Andrej Stancak

**Affiliations:** Department of Psychological Sciences, University of Liverpool, Liverpool L697ZA, UK; Department of Psychological Sciences, University of Liverpool, Liverpool L697ZA, UK; Department of Psychological Sciences, University of Liverpool, Liverpool L697ZA, UK

**Keywords:** activation likelihood estimation, neuroimaging, perception action model

## Abstract

**Background:**

Empathy for pain is a complex phenomenon incorporating sensory, cognitive and affective processes. Functional neuroimaging studies indicate a rich network of brain activations for empathic processing. However, previous research focused on core activations in bilateral anterior insula (AI) and anterior cingulate/anterior midcingulate cortex (ACC/aMCC) which are also typically present during nociceptive (pain) processing. Theoretical understanding of empathy would benefit from empirical investigation of shared and contrasting brain activations for empathic and nociceptive processing.

**Method:**

Thirty-nine empathy for observed pain studies (1112 participants; 527 foci) were selected by systematic review. Coordinate based meta-analysis (activation likelihood estimation) was performed and novel contrast analyses compared neurobiological processing of empathy with a comprehensive meta-analysis of 180 studies of nociceptive processing (Tanasescu *et al.,* 2016).

**Results:**

Conjunction analysis indicated overlapping activations for empathy and nociception in AI, aMCC, somatosensory and inferior frontal regions. Contrast analysis revealed increased likelihood of activation for empathy, relative to nociception, in bilateral supramarginal, inferior frontal and occipitotemporal regions. Nociception preferentially activated bilateral posterior insula, somatosensory cortex and aMCC.

**Conclusion:**

Our findings support the likelihood of shared and distinct neural networks for empathic, relative to nociceptive, processing. This offers succinct empirical support for recent tiered or modular theoretical accounts of empathy.

## Introduction

Empathy is a critical concept in human emotional and social experience. The ability to share in the affective states of those around us brings evolutionary advantages, enabling us to respond to the needs of others, predict their behaviour and support decision-making about our own actions (Decety *et al.,* 2012). Empathy for observed pain leads to the generation of a negative affective or cognitive state which is generally considered to be aversive. However, this process contributes to the benefit of the individual via learning-protective functions (Craig, 2004) and societal groups by inducing prosocial helping behaviours (Hein *et al.,* 2010; Decety *et al.,* 2016). In this fashion, empathy influences our day-to-day personal experience, aspects of mental health and well-being and impacts on societal structures (Bernhardt and Singer, 2012).

Nociceptive (pain) processing is associated with wide-reaching patterns of neural activation which briefly encompass bilateral anterior, mid-and-posterior insula cortices, primary and secondary somatosensory cortex, inferior frontal gyri (IFG) and supramarginal gyri, as well as medial clusters in anterior cingulate/anterior midcingulate cortices (ACC/aMCC), thalami and brainstem (Melzack, 2001; Wager *et al.,* 2013). Previous neuroimaging studies highlighted a similar pattern of neural activation during empathy for pain, with particular overlap between direct experience of pain and empathy for pain described in ACC/aMCC and anterior insula (AI) cortex (Morrison *et al.,* 2004; Singer *et al.,* 2004; Jackson *et al.,* 2005). As such, it was posited that shared representations in the brain for empathy and direct pain experience could underpin the similarities in neural activation profiles for both (Preston and de Waal, 2002; de Vignemont and Singer, 2006).

The Perception–Action Model (PAM) of empathy (Preston and de Waal, 2002), suggests that attended perception of another’s state automatically activates one’s own subjective representations, leading to generation of associated responses unless inhibited. As well as aforementioned neuroimaging studies, research in support of the PAM highlighted the automaticity of empathic responses from humans (Lamm *et al.,* 2007) through to lower order mammals (Langford *et al.,* 2006). Such automaticity may reflect an inherent dependence on specific neurobiological mechanisms e.g. mirror neuron systems (Rizzolatti and Craighero, 2005) which could facilitate the close relationship between perception and action responses.

However, current definitions of empathy suggest the involvement of automatic affective processing but also include aspects of higher order cognition. For example, empathy requires isomorphic sharing of feelings with another, but also necessitates awareness that one’s state originates from observation of the target (Singer and Lamm, 2009). In support of this, neuroimaging research has shown that empathic brain responses in humans are modulated by top–down psychological factors including (but not limited to) situational context (Singer, 2006), intentionality (Akitsuki and Decety, 2009), in/out-group status (Azevedo *et al.,* 2014), implicit bias (Azevedo *et al.,* 2013), pain catastrophising (Fallon *et al.,* 2015) and relevant expertise (Cheng *et al.,* 2007). Top–down modulation of empathic brain responses points to processes beyond mere shared representation, which would likely necessitate a pattern of brain activation that is at least partly distinct from nociceptive pain processing.

The Russian-Doll model of empathy posits a tiered system with progressive levels of empathy from basic affective (e.g. emotional contagion) to higher order processes such as sympathetic concern and emotional perspective taking (de Waal, 2008). Similarly, other proponents also suggest multifaceted neuroscientific theoretical models of empathy which necessitate shared representations between self and other, plus the ability to distinguish between the two, as distinct and essential building blocks for empathic experience (Lamm *et al.,* 2016). Likewise, the most recent iteration of the PAM describes a dynamic and graded system with flexibility for learning and experience which is likely to recruit different brain regions for distinct components of empathic processing (de Waal and Preston, 2017). Novel approaches are required to provide empirical data to support understanding of brain regions or networks of regions involved in shared and distinct theorized components for empathy and direct pain processing.

Neuroimaging studies demonstrate a heterogeneous profile of activation foci for empathy for pain (Zaki *et al.,* 2016), which may be due in part to fundamental issues with sample size, study designs and the mass univariate approach typically employed in fMRI studies (Lamm *et al.,* 2016). Meta-analysis represents a pertinent tool for understanding the social neuroscience of empathy due to its inherent advantages for overcoming issues including design heterogeneity and small sample sizes (Button *et al.,* 2013). Earlier meta-analyses of empathy for pain highlighted a conjunction for direct pain experience and empathy for pain in a core network of brain ACC/aMCC and AI bilaterally (Lamm *et al.,* 2011). Similar regions were also seen in a coordinate based meta-analysis of empathic processing including, but not restricted to, pain (Fan *et al.,* 2011) and empathic processing for empathy which excluded empathy for pain modality (Bzdok *et al.,* 2012).

More recent coordinate based meta-analyses have confirmed core activations for empathy for pain in AI and ACC/aMCC. However, they also revealed additional activations profiles in postcentral gyrus, inferior parietal lobe and deep brain structures including thalamus and brainstem (Timmers *et al.,* 2018) bilateral IFG, supramarginal and fusiform regions and the right anterior lobe of the cerebellum (Jauniaux *et al.,* 2019; Xiong *et al.,* 2019). Given the likelihood of an extended network of empathic processing, it would now be advantageous to consider spatial conjunction and contrast for empathy for pain and processing of actual pain. Although neuroimaging studies have previously considered conjunction (Lamm *et al.,* 2011), until now, there is no meta-analysis of brain imaging research to investigate the spatial contrast between empathy for pain and direct pain experience.

Furthermore, the current interpretation of existing data, which points to overlap of empathy and direct processing for pain in aMCC and AI, is further complicated by the relevance of these regions for broader states that are independent of pain processing such as interoception, arousal and attention (Craig, 2009). Indeed, these regions are statistically the most commonly activated across an expansive range of fMRI experiments with varying themes (Yarkoni *et al.,* 2011). Moreover, the specificity of pain-related activations in AI and aMCC was recently criticized (Iannetti *et al.,* 2013), which in turn casts doubt on the relevance of conjunction activations with empathy for pain. Taken together, we need to consider new empirical approaches which will allow for an improved understanding of how activation profiles for empathy for pain relate to, and differ from, those associated with direct experience of pain to inform our theoretical understanding of empathic processing.

To achieve this aim, the present study proposed to compare brain activations associated with empathy for observed pain and nociceptive processing by employing a coordinate based meta-analysis with activation likelihood estimation (ALE, Eickhoff *et al.,* 2009; Eickhoff *et al.,* 2012). A principle aim of our meta-analysis was to consider the whole-brain network of brain activations associated with empathy for observed brain whilst minimizing bias, we therefore opted to exclude studies which only utilized region of interest (ROI) analysis which may bias meta-analyses towards more established or accepted regions (Eickhoff *et al.,* 2012; Turkeltaub *et al.,* 2012). In addition, we expanded on previous investigations by performing conjunction and contrast analyses of our ALE findings with those of a recent comprehensive coordinate based meta-analysis of pain processing (Tanasescu *et al.,* 2016). This allows, for the first time, an empirical meta-analysis evaluation of shared, and distinct, activation profiles for empathy for pain and direct pain experience.

Following-on from recent extended meta-analyses, we hypothesized that empathy for observed pain would demonstrate a greater degree of overlap with nociceptive pain processing which would extend beyond established core activations in AI and aMCC. In addition, we posited that empathy for pain and direct experience of pain would each demonstrate distinct activation profiles in line with recent theoretical descriptions which highlight the likelihood of modular components of empathy with potential for hierarchical functional relevance (Lamm *et al.,* 2016; de Waal and Preston, 2017). We anticipated that empathy for pain, relative to actual pain experience, would demonstrate increased likelihood of activation in brain regions associated with higher order processing and particularly self-other distinction such as parietal cortices and temporoparietal junction (TPJ). Conversely, it was expected that direct pain experience would elicit greater likelihood of activations in brain regions such as posterior insula which receives afferent nociceptive projections (Garcia-Larrea, 2012) and therefore would not be recruited by empathy for pain.

## Method

### Data search and extraction

The formal search strategy was conducted according to the PRISMA guidelines for reporting meta-analyses and systematic reviews (Moher *et al.,* 2009). This consisted of systematically searching four electronic databases during October 2019 (Medline, Pubmed, PsycINFO, Scopus) using the MeSH search terms (magnetic resonance imaging OR fMRI) AND (functional OR brain activation OR neural activity OR BOLD) AND (Pain) AND (empathy OR empathic). Searches were restricted to terms found in the title or abstract of articles. No date limit was set for the searches and manual searches of the reference sections of identified papers were conducted to supplement the formal search process. Previous meta-analyses of empathic processing (Fan *et al.,* 2011; Lamm *et al.,* 2011; Timmers *et al.,* 2018; Jauniaux *et al.,* 2019) were also screened for additional articles although this did not lead to the inclusion of any further studies.

#### Article selection and extraction of data

Formal database searches were conducted by two authors independently (N.F. and C.R.), as were supplementary and manual searches. Both authors were responsible for assessment of articles for inclusion, and decisions over article inclusion were determined by discussion, disagreements where resolved via discussion or presented to a third arbiter (A.S.). One author (N.F.) extracted the relevant coordinate data, which was cross-checked and confirmed by a second (C.R.). Studies that reported coordinates in the Talairach space were converted into MNI using GingerALE software for the purposes of analysis and reporting.

#### Eligibility criteria

The criteria for inclusion were: (i) human fMRI studies published up until October 2019; (ii) original English language articles; (iii) published in a peer-reviewed journal; (iv) utilizing a paradigm including visual pain stimuli i.e. images, videos or animations of pain scenes or pain facial expressions; (v) employed an appropriate contrast with a suitable control stimulus (e.g. non-painful scene, animation, etc.); (vi) coordinates were reported in the paper or supplementary material of the direct (pain > non-pain) contrast in either Montreal Neurological Institute (MNI, (Evans *et al.,* 1993)) or Talairach space, studies that reported coordinates in the Talairach space were converted into MNI for the purposes of analysis and reporting; (vii) data were obtained from a healthy population without experience or expertise that could modulate empathic processing (e.g. we excluded studies that explicitly recruited only clinicians or other healthcare professionals or only incarcerated individuals); (viii) whole-brain analysis were reported with thresholding of (or equivalent to) *P* < 0.001 uncorrected voxelwise throughout the whole brain with at least *P* < 0.05 cluster level correction (or equivalent) declared. We excluded papers which only reported ROI results.

Meta-analysis has shown that the selected level of hierarchical thresholding is optimal for balancing sensitivity to effect with reduced risk of false positives (Woo *et al.,* 2014). This level was selected to reduce bias by reducing the inclusion of false positive reports which are highly likely to favour specific regions that are established in the literature. This was also the reason for excluding studies which only utilized ROI analyses. In few potentially acceptable studies when no thresholding was reported, we contacted individual groups to request details but received no replies. For comparison with direct pain experience, we utilized an existing open-access database of coordinates included in a previous meta-analysis of experimental pain stimulation during fMRI (Tanasescu *et al.,* 2016). Inclusion criteria were similar to those in the present study. Briefly, the authors only included research that reported whole-brain group analysis of a pain induction>baseline contrast. From the full dataset (which included subgroups of chronic pain populations and also healthy people with chemically induced hyperalgesia) we extracted only studies which focused on cutaneous pain in healthy populations to reflect the population extracted for the present empathy meta-analysis. Further details on the studies included can be found in Supplementary Materials 3.

In order to determine consistency in reported regions of activation for both analyses, ALE meta-analyses was performed in Brainmap GingerALE v2.3.6 (Eickhoff *et al.,* 2009). This method assigns an ALE value to each voxel throughout the whole brain, with greater ALE values indicative of more studies which report activated peaks at a voxel or in close proximity. We implemented the more stringent correction of ALE estimation (Turkeltaub *et al.,* 2012). This method utilizes a random effects model to minimize within-experiment effects (by accounting for the number and proximity of reported foci) and within-group effects (by accounting for multiple contrasts reported from a single study), both of which can unduly influence meta-analyses. This method therefore optimizes the degree to which ALE values represent concordance of findings across independent contributions.

Standardized procedures and default parameters were employed throughout the analysis adhering to recent guidelines on methodology for appropriate study selection and thresholding of results (Eickhoff *et al.,* 2016). The concordance of ALE values throughout the brain for empathy for pain (empathy > control), and direct processing of pain (pain > non-pain) were evaluated in comparison to random distributions using permutation analysis (Maris and Oostenveld, 2007) with 10 000 permutations. Deactivation contrasts (e.g. control > empathy, non-pain > pain) were not included due to the infrequency of reporting of this direction of contrast in the literature. An initial cluster forming threshold (uncorrected *P* < 0.001) was implemented followed by cluster-level Family-wise error (FWE) correction (*P* < 0.05) to identify relevant ALE regions as recommended in recent publications (Eickhoff *et al.,* 2016). Secondly, resulting ALE maps for empathy for pain and direct pain experience were compared using conjunction and contrast analyses. Again permutation analysis was performed with 10 000 permutations to identify brain regions which demonstrated overlapping and distinct patterns in the respective ALE maps for each process. For cluster analysis, results were thresholded using cluster-level false discovery rate (FDR, *P* < 0.05) and a minimum cluster size of 200 mm^3^ as previously recommended (Eickhoff *et al.,* 2016). FDR correction is the most stringent correction method available in Ginger ALE software for contrast analyses, FWE method is not available for contrast analyses (Hoffman and Morcom, 2018; Papitto *et al.,* 2020).

**Fig. 1:**
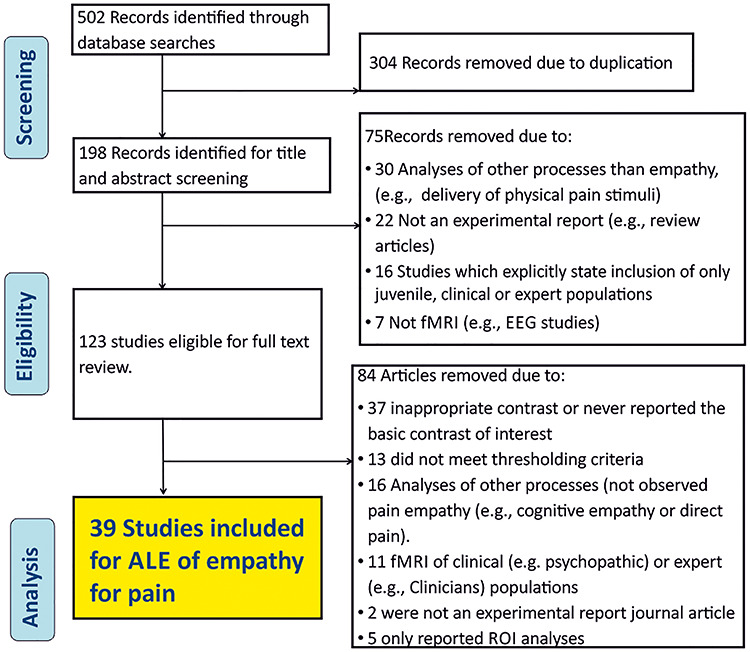
Flow chart depicting the initial search and eligibility screening process.

## Results

Figure 1 illustrates a flowchart indicating the study selection steps, (see Supplementary Material for spreadsheet of search process and PRISMA checklist). Specifically, a total of 502 articles were returned from the initial searches (Medline; 124; Pubmed; 154, PsycInfo; 129, Scopus; 95). Of these, 304 were duplicates from repeated searches and removed in the first step. A further 75 articles were removed following the initial review of titles and abstracts. Studies excluded at this stage included: those where it was clear and obvious that no suitable (i.e. healthy adult) population was reported (16), studies where it was clear and obvious that they did not utilize observed pain stimuli (30), not an experimental report (e.g. review articles) (22), not fMRI method (7). Furthermore, following full-text review a further 84 articles were removed including those which exhibited an inappropriate contrast (e.g. comparisons that did not match the selection criteria, in-group empathy > out-group empathy) or never reported the basic contrast of interest (empathy > control) (37), those which never met the thresholding criteria (13), did not utilize observed pain stimuli (16), never reported a healthy, adult and non-expert population (11), were not an experimental report journal article (2) or which only reported ROI analyses (5), leaving a total of 39 studies for the analyses of empathy for pain (Table 1).

**Table 1:** Studies included in ALE meta-analysis of empathy for pain

Author	Year	Title	N	Mean age (SD)	Description of empathy stimuli
Akitsuki *et al.*	2009	Social context and perceived agency affects empathy for pain: an event-related fMRI investigation	26	28.9 (5.6)	Animations of feet and hands in pain
Azevedo *et al*.	2013	Their pain is not our pain: brain and autonomic correlates of empathic resonance with the pain of same and different race individuals	27	23.57 (4.01)	Videos of hands inserted with needles
Azevedo *et al.*	2014	Weighing the stigma of weight: an fMRI study of neural reactivity to the pain of obese individuals	12	22.2 (2.6)	Video of faces inserted with needles
Benuzzi *et al.*	2018	Pain mirrors: neural correlates of observing self or others’ facial expressions of pain	27	21.3	Videos depicting painful facial expressions
Berlingeri *et al.*	2016	Guess who’s coming to dinner: brain signatures of racially biased and politically correct behaviours	25	25.3 (4.81)	Videos of hands inserted with needles
Bos *et al.*	2015	Oxytocin reduces neural activity in the pain circuitry when seeing pain in others	24	23.1	Videos of hands inserted with needles
Cao *et al.*	2015	Racial bias in neural response to others’ pain is reduced with other-race contact	30	23.17 (1.8)	Video of faces inserted with needles
Cao *et al.*	2019	Neural and behavioural markers of observed pain of older adults	29	21.42	Videos of faces with needles inserted
Cheng *et al.*	2010	Love hurts: an fMRI study	36	23 (3)	Animations of feet and hands in pain
Christov-Moore *et al.*	2019	Sex differences in somatomotor representations of others’ pain: a permutation-based analysis	70	18–35	Videos of hands with needles inserted
Contreras-Huerta *et al.*	2013	Racial bias in neural empathic responses to pain	20	22.5 (1.06)	Video of faces inserted with needles
Enzi *et al.*	2016	Empathy for pain-related dorsolateral prefrontal activity is modulated by angry face perception	20	27 (5.08)	Picture of faces or hands with needles inserted
Fan *et al.*	2014	Empathic arousal and social understanding in individuals with autism: evidence from fMRI and ERP measurements	21	19.3 (3.4)	Images of hands and feet in painful situations
Feng *et al.*	2016	Social hierarchy modulates neural responses of empathy for pain	22	22.23 (1.85)	Picture of faces or hands with needles inserted
Fourie *et al.*	2017	Empathy and moral emotions in post-apartheid South Africa: an fMRI investigation	38	40.11 (4.12)	Facial expressions of pain
Gu *et al.*	2013	Cognition-emotion integration in the anterior insular cortex	18	25.2	Pictures hands feet painful situation
Guo *et al.*	2012	Empathic neural responses to others’ pain depend on monetary reward	16	23.5	Images depicting hand, fingers, ears in painful situations
Guo *et al.*	2013	Exposure to violence reduces empathetic responses to other’s pain	40	22.15 (2.67)	Images depicting hand, fingers, ears in painful situations
Han *et al.*	2017	Empathy for pain motivates actions without altruistic effects: evidence of motor dynamics and brain activity	33	22.91 (2.47)	Video of faces with needles inserted
Jackson *et al.*	2006	Empathy examined through the neural mechanisms involved in imagining how I feel *vs* how you feel pain	34	29 (6.5)	Images of hands and feet in painful situations
Jackson *et al.*	2005	How do we perceive the pain of others? A window into the neural processes involved in empathy	15	22 (2.6)	Images of hands and feet in painful situations
Krach *et al.*	2015	Evidence from pupillometry and fMRI indicates reduced neural response during vicarious social pain but not physical pain in autism	16	24.3	Images of hands and feet in painful situations
Lamm & Decety.	2008	Is the extrastriate body area (EBA) sensitive to the perception of pain in others?	18	23.67 (3.99)	Images of hands with needles inserted
Laselle *et al.*	2019	Influence of anxiety and alexithymia on brain activations associated with the perception of others’ pain in autism	20	24.15	Videos of hands or feet with needle or scalpel
Ma *et al.*	2011	Neural responses to perceived pain in others predict real-life monetary donations in different socioeconomic contexts	33	22.4 (2)	Videos of faces or hands with needles inserted
Majdandzic *et al.*	2016	The selfless mind: how prefrontal involvement in mentalizing with similar and dissimilar others shapes empathy and prosocial behaviour	32	22.7 (3.2)	Videos of people receiving painful shocks
Morelli *et al.*	2014	The neural components of empathy: predicting daily prosocial behaviour	32	19.9 (1.4)	Images of hands and feet in painful situations
Noll-Hussong *et al.*	2013	Neural correlates of deficits in pain-related affective meaning construction in patients with chronic pain disorder	19	46.62 (12.49)	Images of hands and feet in painful situations
Preis *et al.*	2013	The effects of prior pain experience on neural correlates of empathy for pain: an fMRI study	64	22.98 (4.1)	Images depicting hands receiving pressure pain from an algometer
Richins *et al.*	2019	Empathic responses are reduced to competitive but not non-competitive outgroups	69	20.57	Pictures hands feet painful situation
Ruckmann *et al.*	2015	How pain empathy depends on ingroup/outgroup decisions: a functional magnet resonance imaging study	30	24.5 (3.36)	Images of hands and feet in painful situations
Seara-Cardoso *et al.*	2015	Neural responses to others’ pain vary with psychopathic traits in healthy adult males	46	27. 93	Images of hands and feet in painful situations
Sheng *et al.*	2014	Task modulations of racial bias in neural responses to others’ suffering	21	22 (1.8)	Facial expressions of pain
Vachon-Presseau *et al.*	2012	Neural processing of sensory and emotional-communicative information associated with the perception of vicarious pain	20	36 (10)	Images depicting hand, feet in pain situations or facial experssions of pain
van der Heiden *et al.*	2013	Inter-individual differences in successful perspective taking during pain perception mediates emotional responsiveness in self and others: an fMRI study	18	25.3(2.54)	Images depicting hand, feet in pain situations
Vistoli *et al.*	2016a	Changes in visual perspective influence brain activity patterns during cognitive perspective-taking of other people’s pain	21	29.2 (7.9)	Animations depicting hands in pain situations
Wang *et al.*	2015	Challenging emotional prejudice by changing self-concept: priming independent self-construal reduces racial in-group bias in neural responses to other’s pain	30	22.6 (2.4)	Videos of faces or hands with needles inserted
Zheng *et al.*	2016a	Perceived reputation of others modulates empathic neural responses	20	25 (1.6)	Images depicting hands or fingers in painful situations
Zheng *et al.*	2016b	Decreased empathic responses to the ‘lucky guy’ in love: the effect of intrasexual competition	20	21.7 (1.89)	Images depicting hand, fingers, ears in painful situations

### Significant ALE clusters for empathy for pain.

**Fig. 2:**
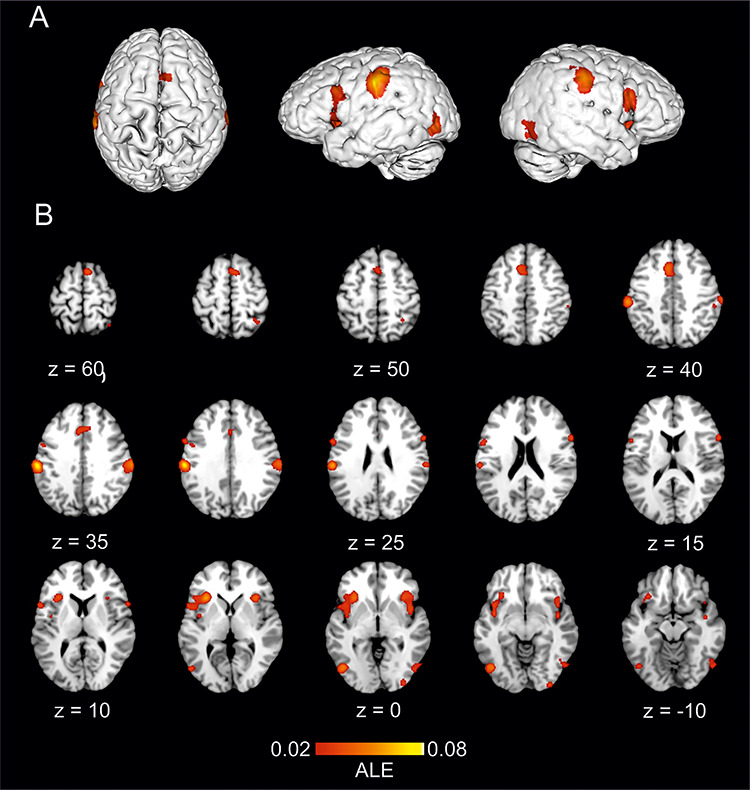
The location of significant ALE clusters from the meta-analysis of concordant activations for empathy for pain. Results are displayed overlaid onto a standardized MNI template anatomical brain in (A). 3D surface projection. (B) As a montage of coronal slices throughout the whole brain. ALE scores are indicated by colourbar.

The empathy for observed pain–non-pain contrast ALE meta-analysis pooled data from a total of 1112 participants and 527 reported foci. The results (Table 2) revealed 10 significant clusters where ALE values demonstrated significant levels of consistent spatial activation for empathy for observed pain. Two large bilateral clusters covered AI extending laterally to IFG with a smaller independent cluster also evident in right IFG. Bilateral clusters were identified in supramarginal and lateral occipitotemporal cortices and a medial cluster was elicited in ACC/aMCC extending superiorly to supplementary motor cortex. Figure 2 illustrates the location of significant ALE clusters from the meta-analysis of empathy for pain.

**Table 2:** Locations of significant clusters from the ALE map of empathy for pain

Cluster	Label	Volume (mm^3^)	*x*	*y*	*z*	# Studies	ALE peak
1	Left anterior insula	9800	-30	22	4	34	0.054
	Left inferior frontal gyrus		-58	10	28		0.030
	Left anterior insula		-40	2	-4		0.029
	Left anterior insula		-40	12	-4		0.028
	Left precentral gyrus		-54	12	8		0.028
	Left inferior frontal gyrus		-54	8	20		0.026
	Left precentral gyrus		-50	4	34		0.22
	Left anterior insula		-40	-2	12		0.019
2	Anterior midcingulate cortex	5800	-6	18	40	23	0.040
	Supplementary motor cortex		6	14	60		0.032
	Anterior cingulate cortex		6	26	34		0.026
	Anterior midcingulate cortex		2	18	28		0.020
3	Left supramarginal gyrus	3232	-58	-22	34	22	0.075
4	Right anterior insula	4192	32	22	4	22	0.037
	Right anterior insula		42	8	0		0.029
	Right anterior insula		42	24	-2		0.028
	Right claustrum		42	-2	-6		0.027
5	Right supramarginal gyrus	3232	64	-22	36	13	0.046
	Right supramarginal gyrus		54	-32	44		0.018
6	Right lateral occipitotemporal cortex	2496	-44	-68	-2	14	0.048
7	Right lateral occipitotemporal cortex	1720	52	-66	-10	11	0.023
	Right lateral occipitotemporal cortex		46	-60	-6		0.019
8	Right middle frontal gyrus	1448	58	14	22	8	0.030
9	Right occipital cortex	760	32	-90	-4	6	0.028
10	Right superior parietal lobule	736	32	-50	52	5	0.021

### Directly perceived pain

The findings of the coordinate based analysis for directly perceived pain studies reflect the results from the existing research where they are described, illustrated and discussed in more detail (Tanasescu *et al.,* 2016). To briefly summarize, our ALE meta-analysis contrast of experimental pain in healthy participants—baseline pooled data from 180 eligible studies with a total of 2605 participants and 2780 reported foci. Results revealed four (extensive) clusters of activation encompassing primary and secondary somatosensory cortices (S1, S2), bilateral anterior, mid and posterior insula, prefrontal and premotor cortices, bilateral putamen and thalami and medial clusters in ACC/aMCC and brainstem regions.

### Conjunction analysis

**Fig. 3:**
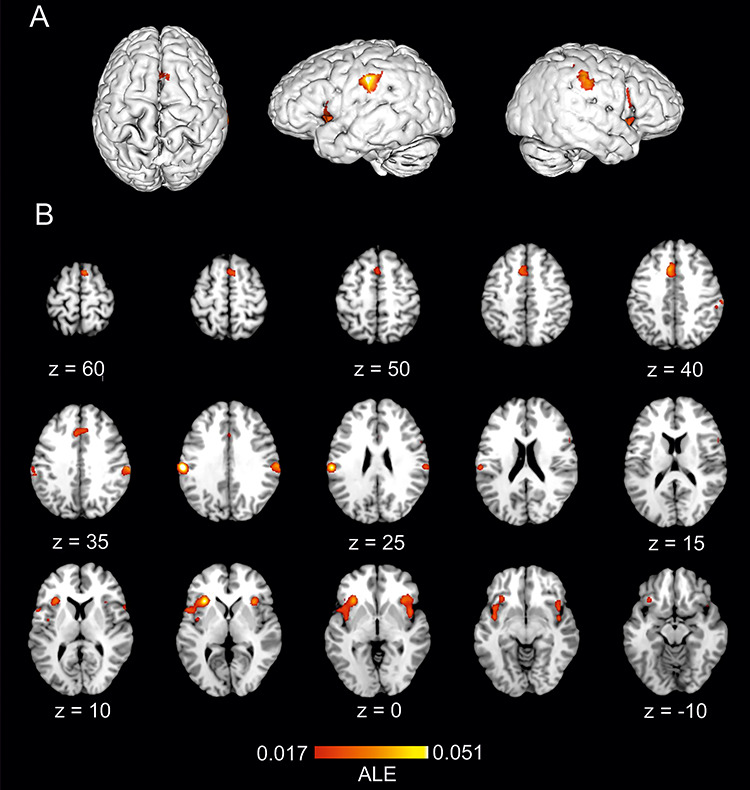
The location of significant clusters from conjunction analysis of ALE maps for empathy for pain and directly perceived pain. Results are displayed overlaid onto standardized MNI template anatomical brain in (A). 3D surface projection. (B) As a montage of coronal slices throughout the whole brain. ALE scores are indicated by colourbar.

The conjunction analysis of ALE maps representing empathy for pain and direct pain experience pooled data from 219 studies with a total of 3717 participants and 3307 reported foci. The results highlighted an overlap of activation likelihood coordinates in seven clusters encompassing bilateral AI and ACC/aMCC as well as bilateral IFG which bordered (and in the case of the right hemisphere extended to) middle frontal gyrus, and bilateral supramarginal regions (Table 3, Figure 3).

**Table 3:** Locations of significant clusters from conjunction analysis of empathy for pain and directly perceived experimental pain

Cluster	Label	Volume (mm^3^)	*x*	*y*	*z*	ALE peak
1	Left anterior insula	7096	-30	22	4	0.045
	Left anterior insula		-40	2	-4	0.028
	Left inferior frontal gyrus		-54	12	8	0.028
	Left anterior insula		-40	12	-4	0.028
	Left inferior frontal gyrus		-50	10	4	0.027
	Left orbitofrontal cortex		-32	24	-10	0.021
	Left mid insula		-40	-2	12	0.019
2	Anterior midcingulate cortex	4920	-6	18	40	0.040
	Supplementary motor cortex		6	14	60	0.031
	Anterior cingulate cortex		6	26	34	0.026
	Anterior midcingulate cortex		2	18	28	0.019
3	Right anterior insula	4008	34	22	4	0.037
	Right anterior insula		42	8	0	0.029
	Right anterior insula		42	24	-2	0.028
	Right claustrum		42	-2	-6	0.026
4	Left supramarginal gyrus	2376	-60	-22	30	0.065
5	Right supramarginal gyrus	2256	62	-24	36	0.040
	Right supramarginal gyrus		54	-32	44	0.018
6	Right middle frontal gyrus	416	56	14	22	0.024
	Right inferior frontal gyrus		58	14	12	0.022
	Right inferior frontal gyrus		56	14	16	0.022

### Contrast analyses: empathy—pain

**Fig. 4:**
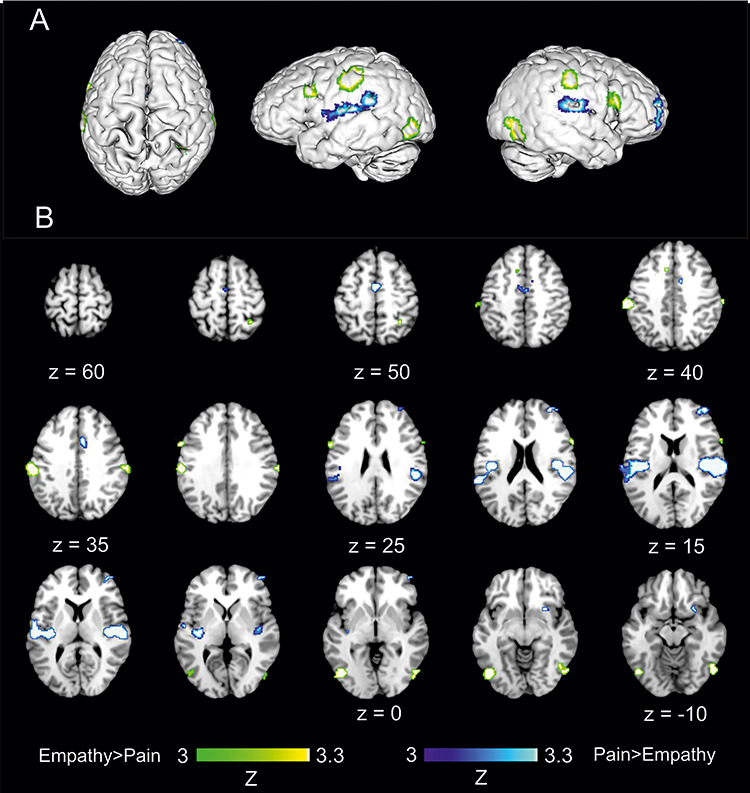
The location of significant clusters from contrast analysis of ALE maps for greater likelihood of empathy for pain relative to directly perceived pain and vice versa. Results in green indicate regions which showed greater likelihood of concordance of activation for empathy for pain, but not for direct pain experience. Results in blue indicate regions which showed greater likelihood of concordance of activation for direct pain experience, but not for empathy for pain. All clusters are overlaid onto standardized MNI template anatomical brain in (A). 3D surface projection. (B). As a montage of coronal slices throughout the whole brain. Relative Z scores are indicated by colourbar.

Contrast analysis comparing the ALE maps of concordant activations for each process pointed to significantly greater likelihood of activation during empathy for pain relative to directly perceived pain in 6 clusters encompassing bilateral supramarginal, IFG and occipitotemporal regions (Table 4, Figure 4).

### Contrast analyses: pain—empathy

The reverse contrast revealed six clusters indicative of increased activation likelihood estimates for directly perceived pain relative to empathy for observed pain. These regions encompassed two large bilateral clusters encompassing parietal opercular cortices (S2), posterior insula and S1. Right putamen was also evident for directly perceived pain relative to empathy. A right frontal cluster encompassing right prefrontal and dorsolateral prefrontal cortices was also elicited. Two medial clusters demonstrated increased concordance of activation in direct, relative observed, pain in aMCC (Table 5, Figure 4).

## Discussion

The findings of the ALE meta-analysis of empathy for pain revealed concordant activations to observed pain stimuli located in aMCC and bilateral AI which accords with previous investigations (Fan *et al.,* 2011; Lamm *et al.,* 2011). However, the present results expand on previous analyses to reveal a richer and more complex pattern of activation encompassing bilateral supramarginal, IFG and occipitotemporal cortices. It is noteworthy that previous research did allude to the potential for a broader range of contributing brain regions including some of these specific regions (Lamm *et al.,* 2011). Moreover, such a restricted profile of activation is inconsistent with the subjective complexity of empathic experience and the heterogeneity of activation foci seen across relevant fMRI studies (Zaki *et al.,* 2016). Therefore, the present analysis aligns with recent meta-analyses to confirm the existence of previously established core activations in AI and aMCC (Fan *et al.,* 2011; Lamm *et al.,* 2011; Bzdok *et al.,* 2012) for empathic processing, but also indicates the likelihood of additional activations in bilateral inferior parietal lobe, IFG and occipitotemporal cortices (Timmers *et al.,* 2018; Jauniaux *et al.,* 2019; Xiong *et al.,* 2019). Taken together, this body of research goes some way towards establishing neurobiological underpinnings of empathy by highlighting the involvement of an extended network beyond these core brain regions. In the present analysis, all brain regions described in results were significant contributors to the ALE of empathy for pain after implementing stringent and recently recommended guidance regarding statistical thresholding (Turkeltaub *et al.,* 2012; Eickhoff *et al.,* 2016; Eickhoff *et al.,* 2017) and study selection (Moher *et al.,* 2009; Woo *et al.,* 2014). Moreover, left supramarginal gyrus was amongst the most frequently observed regions in terms of contributing studies, even surpassing right AI, which suggests that the accepted predominance of core regions does not truly reflect the neurobiological underpinning of empathy for observed pain.

The present study was the first to utilize coordinate based meta-analytical methods to statistically evaluate the conjunction and contrast between empathy for observed pain and direct painful experience. For this aim, we compare ALE maps of empathy for pain with those of a conclusive analysis of nociceptive processing comprising some 180 experimental pain studies in healthy people (Tanasescu *et al.,* 2016). Similar to the extended pattern of foci observed for empathy for pain, conjunction analysis highlighted a broad range of overlap for empathy for pain and direct experience of pain with bilateral clusters in AI, IFG and supramarginal gyri, as well as medial clusters in ACC/aMCC. These regions are all important for pain processing, and highlighted in the recent fMRI neural signature of pain (Wager *et al.,* 2013) and its predecessor, the pain neuromatrix (Melzack, 2001). The conjunction between pain and empathic processing builds upon previous image-based analyses which suggested that spatial overlap was primarily limited to AI and aMCC, although this previous study exclusively utilized cue-based, as opposed to visual observation of pain, paradigms (Lamm *et al.,* 2011). The present results go further and support proponents of the PAM (Preston and de Waal, 2002; de Waal, 2008; de Waal and Preston, 2017) by demonstrating a broader functional network of brain regions which occupy the same morphometric space and are recruited during observed pain phenomena and direct experience of pain. This provides empirical support for theoretical interpretations which recognize a degree of shared representation between empathy and direct experience of pain (Singer and Lamm, 2009; Bernhardt and Singer, 2012; de Waal and Preston, 2017; Lamm *et al.,* 2017). In summary, we believe that the present meta-analysis represents the most succinct evidence to date of an extensive conjunction for pain experience and empathy for pain, encompassing a diverse array of pain relevant brain regions in a bilateral pattern indicative of a coherent network. However, it is important to note that evidence of conjunction of processing between nociceptive and empathic pain does not necessarily imply the same psychological representation (Zaki *et al.,* 2016). For example, debate around functional specificity and complexity for pain (and associated) processing in anterior cingulate region is well documented (Lieberman and Eisenberger, 2015; Wager *et al.,* 2016).

Contrast ALE analyses to compare concordant activations that exhibited greater likelihood for empathy for pain compared to direct experience of pain (and vice versa) were also performed for the first time. Empathy, compared to direct pain experience, demonstrated preferential bilateral activation in supramarginal regions, which extended superiorly to the supramarginal gyrus. Although supramarginal activations are frequently reported in fMRI empathy literature, their specific relevance is often not subject to discussion or interpretation, particularly if the basic empathy contrast is not the primary aim of the research (Jackson *et al.,* 2006; Guo *et al.,* 2013; Fan *et al.,* 2014; Berlingeri *et al.,* 2016; Zheng *et al.,* 2016; Benuzzi *et al.,* 2018). Of the many studies contributing supramarginal activations to the present ALE analysis, few discussed their relevance. Those which did interpreted the activation in terms of understanding and anticipating pain (Azevedo *et al.,* 2013), or appraising unpleasantness (Lamm *et al.,* 2007). Others designated this activation as S2, and proposed a more sensory role (Ma *et al.,* 2011). The consistency of bilateral activation of supramarginal regions for empathy suggests a need for greater understanding of its role. fMRI research has demonstrated that supramarginal activity can represent recognition of noxious environmental stimuli (Benuzzi *et al.,* 2008) and reorientation of attention to threat (Decety and Lamm, 2007; Carter and Huettel, 2013). This potentially offers a mechanistic explanation of activity in supramarginal gyrus during empathy for observed pain stimuli, which could underpin the previously proposed higher order interpretations regarding anticipation or valence appraisal (Lamm *et al.,* 2007; Azevedo *et al.,* 2013) which would be required for identification and orientation to threat.

Contrast analyses also pointed to bilateral occiptotemporal activations that were more likely to be engaged during observed pain, but not direct pain experience. Previous fMRI research have explained these activations for empathic viewing in terms of enhanced visual processing (Azevedo *et al.,* 2013) likely due to augmented salience (Akitsuki and Decety, 2009) or attention (Han *et al.,* 2017) for pain scenes. As with supramarginal activation patterns, such lateral occipitotemporal activations are often reported with minimal or no discussion (Vachon-Presseau *et al.,* 2012; Morelli *et al.,* 2014; Seara-Cardoso *et al.,* 2015; Enzi *et al.,* 2016; Fourie *et al.,* 2017; Richins *et al.,* 2019), despite often being amongst the strongest research results in terms of statistical significance. Lateral occipitotemporal cortex encompasses the extrastriate body (EBA) which is associated with perception of body parts (Downing *et al.,* 2001). As the present meta-analysis which focused on paradigms which included visual depictions of pain scenes, most often to limbs or face, this seems reasonable. However, all studies included used a suitable visual control, i.e. same body part in absence of pain, which suggests some empathy (or at least salience-related) function underlying these augmented bilateral activations during empathic processing. Interestingly, activation in lateral occipitotemporal cortex, and particularly EBA, were previously shown to reflect in-group status during empathy for pain (Azevedo *et al.,* 2013) although another study did not report empathy activation in EBA (Lamm and Decety, 2008). Recent research has demonstrated relevance of EBA for self-other recognition of body parts (De Bellis *et al.,* 2017), which suggests potential for the region in higher order aspects of empathic processing, particularly when observing visual depictions of others undergoing somatic pain. The present findings suggest that the occurrence of activation in these regions is frequent, but not assured. Occipitotemporal activations were identified in 30–40% of fMRI research with no clear pattern suggesting that the activation relates to a specific paradigm or stimuli type. Therefore, the nature of lateral occipitotemporal processing during empathy for pain warrants further investigation.

**Table 4:** Locations of significant clusters from contrast analysis of empathy—pain

Cluster	Label	Volume (mm^3^)	*x*	*y*	*z*	Extrema (*z*)
1	Left supramarginal gyrus	2248	-59	-23	36	3.29
2	Left lateral occipitotemporal cortex	1904	-45	-69	-3	3.29
3	Right lateral occipitotemporal cortex	1400	50	-66	-6	3.29
4	Right supramarginal gyrus	648	62	-21	35	3.29
5	Left inferior frontal gyrus	320	-59	10	27	3.29
6	Right inferior frontal gyrus	216	61	14	19	3.29

Contrast analyses also revealed greater bilateral activation in ventral IFG during empathy for pain relative to direct experience of pain. The IFG is frequently activated during motor imagery or action observation type paradigms (Hétu *et al.,* 2013). As IFG is a core component of the mirror neuron system (Rizzolatti and Craighero, 2005), it has been easy to dismiss this as automatic, perception-action processing and a neighbouring part of this region was also highlighted by conjunction analysis. However, the contrast analysis significance of bilateral IFG in the empathy, relative to pain, contrast analysis suggests the likely importance for empathic processing. Previous studies demonstrated increased activation in IFG when participants were rating observed pain rather than a distractor task leading to the suggestion IFG could also contribute to higher order cognitive processes such as attaching meaning to the empathic situation (Gu and Han, 2007; Budell *et al.,* 2010). Moreover, a recent empathy viewing study identified this region as relevant for the process of mentalizing similarity between oneself and the target that one is observing (Majdandzic *et al.,* 2016). Therefore, our findings suggest that processing of visually observed somatic pain in another could recruit activation of IFG to facilitate elements of action-understanding and self-other processing.

ALE of directly perceived pain, compared to empathy, demonstrated preferential concordance of activation in bilateral S1, posterior insula and parietal operculum, right putamen, right prefrontal cortices and aMCC. Posterior insula and parietal opercular cortices represent the primary targets of nociceptors in the spinothalamic tract (Garcia-Larrea, 2012) and the medial regions highlighted in the contrast reflect functional imaging of somatic aspects of pain perception such as gauging pain intensity (Coghill *et al.,* 1999). Overall the contrast points to an absence of empathic processing in medial pain-processing regions, suggesting a pattern wherein empathy for pain shares more overlap with cognitive-affective aspects than somatic processing, as was previously hypothesized (Singer *et al.,* 2004), albeit in a broader neural network than previously thought.

From a theoretical perspective, the patterns of ALE seen in conjunction and contrast analyses show alignment with a tiered theoretical understanding of empathic processing such as the Russian-Doll model (de Waal, 2008; de Waal and Preston, 2017) or independent components of shared representation and self-other distinction (Lamm *et al.,* 2016). The conjunction of activations in bilateral pain processing brain regions suggests a degree of automatic state-matching or emotional contagion (Preston and de Waal, 2002; de Waal, 2008). This is extended by empathy distinct activity in supramarginal, occipitotemporal and IFG regions which may reflect higher order aspects of empathy, and particularly mechanisms of self-other distinction. Previously, TPJ was highlighted for distinction of self-other during empathic processing (de Waal and Preston, 2017). Although our analysis did not indicate TPJ activations during empathy, this region shows greatest functional and anatomical connectivity with supramarginal gyrus and occiptotemporal cortices (Carter and Huettel, 2013; Igelström and Graziano, 2017). Previously, it was posited that TPJ activation could be associated with nuances of the empathic situation, particularly when it requires cognitive perspective-taking (de Waal and Preston, 2017). The present meta-analysis utilized paradigms eliciting empathy via observation of pain, which are less dependent on cognitive perspective-taking. Therefore, supramarginal and lateral occipitotemporal activations, which border TPJ, may reflect more automatic aspects of self-other distinction. Possibly reflecting mechanistic processes relevant to self-other distinction including reorienting attention and quantifying threat (Decety and Lamm, 2007; Carter and Huettel, 2013) or interpreting self-other distinction of perceived body parts (De Bellis *et al.,* 2017). We can speculate that the network of regions highlighted by the present, meta-analysis of empathy for observed pain is more relevant to these mechanistic aspects of empathic processing, whereas higher order cognition, such as emotional perspective taking, may recruit TPJ.

**Table 5:** Locations of significant clusters from contrast analysis of pain—empathy

Cluster	Label	Volume (mm^3^)	*x*	*y*	*z*	Extrema (*z*)
1	Right parietal operculum	7728	50	-20	16	3.29
2	Left parietal operculum	6208	-48	-21	14	3.29
3	Right dorsolateral prefrontal cortex	1120	38	53	13	3.29
4	Anterior midcingulate cortex	696	0	-4	50	3.29
5	Anterior midcingulate cortex	408	7	10	36	3.29
6	Right putamen	240	26	13	-7	3.29

The role of inferior parietal and occipitotemporal cortices for a broad range of social processing is a topic of considerable research, and a nexus of social processing extending from the angular gyrus of the TPJ anteriorly to supramarginal gyrus and posteriorly to occipitotemporal cortices was posited (Carter and Huettel, 2013). Others have highlighted the spatial relevance of TPJ and surrounding regions including supramarginal gyrus for a broad range of social cognition and self-other distinction (Igelström and Graziano, 2017). In light of the present meta-analysis, the pattern of activation in these parietal-occipital regions extending beyond the TPJ, which are often neglected in existing functional imaging research, could actually have important relevance for empathy for pain.

The present study has some limitations. As mentioned, we focused on empathy for observed pain rather than more complex iterations of cognitive empathy such as paradigms which utilize learning to associate abstract cues with pain stimulation delivered to another person located outside of the scanner. The former design is more prevalent in fMRI research (Lamm and Majdandžić, 2015), allowing for a greater degree of studies to be included. To include cognitive empathy paradigms would necessitate the permission of a smaller cohort of studies, falling below the minimum 17 independent studies recommended for ALE analysis (Eickhoff *et al.,* 2016). Else it would mean permitting less stringent search and analysis methods which would sacrifice the integrity of the aims which focused on concordance across studies which utilized whole-brain analyses and robust statistical thresholding to reveal empathy for pain activations regardless of existing bias. However, it should be noted that previous meta-analyses suggest distinct patterns of activation for observed pain compared to more cognitive methods of evoking empathic response (Lamm *et al.,* 2011) and specifically that observing visual pain in one’s environment may be more associated with brain regions that are associated with affective and motor-motivational processes (de Waal and Preston, 2017). Furthermore, other factors such as the bodily location of pain, (e.g. face vs. limb) or laterality were not considered, but their importance has been demonstrated in previous meta-analyses (Jauniaux *et al.,* 2019). On the other hand, the decision to include studies which depicted facial expressions of persons experiencing pain, rather than solely observation of pictures or video clips of directly delivered pain stimuli, could add an element of cognitive interpretation (Jauniaux *et al.,* 2019; Xiong *et al.,* 2019) which could also introduce some variance into the meta-analysis and should be considered as a limitation. Therefore, the present analysis should primarily be interpreted in relation to empathy for observation of pain through visual modality, and as a novel comparison with direct pain processing.

To surmise the impact of the present study, elucidation of a rich functional brain network of empathy for pain, extending beyond AI and aMCC, is important for theoretical understanding of the phenomenon. The conjunction and contrast analyses reveal, for the first time, shared and distinct networks for observed and direct pain which supports the concept of tiered levels of processing of empathy as were previously theorized (de Waal, 2008; Bernhardt and Singer, 2012; Lamm *et al.,* 2016; de Waal and Preston, 2017). The findings indicate that an extended network of brain regions warrant greater focus and consideration for their role in empathic processing, particularly regions neighbouring TPJ in inferior parietal and occipitotemporal cortices. Such a broader approach would accord with recent developments and understanding from social neurosciences (Carter and Huettel, 2013; Igelström and Graziano, 2017). Finally, greater understanding of neurobiological underpinnings of empathy has practical implications for diagnosis of clinical disorders as well as therapeutic or pharmacological interventions (Decety *et al.,* 2016; Christov-Moore and Iacoboni, 2019), or development of strategies to promote prosocial behaviours or establish moral, fair and practical societal structures (Decety and Cowell, 2015).

To conclude, the findings reveal concordance in an extensive bilateral network of brain regions for empathy for observed pain. This encompassed bilateral AI, supramarginal gyri, lateral occipitotemporal cortices IFG and aMCC, regions with functional relevance for interoception, pain processing, social cognition and self-other distinction. Utilizing novel contrast analyses for empathy for pain and direct pain experience, we demonstrated a broad network of shared brain representations which align to automaticity of response or emotional contagion, and empathy-specific activation patterns with relevance for higher order responses such as self-other distinction. Knowledge of these shared and distinct brain networks offers a novel insight into the neurobiological underpinnings of our subjective experience of empathy with relevance for theoretical, clinical and social applications.

## Conflict of interest

None declared.

## Supplementary Material

File013_nsaa090
