# Supplementary material for: Shared and distinct functional networks for empathy and pain processing: a systematic review and meta-analysis of fMRI studies
Source: Soc Cogn Affect Neurosci. 2020 Jun 29;15(7):709–23. doi: 10.1093/scan/nsaa090 (PMC7511882; doi:10.1093/scan/nsaa090)
Supplement: File013_nsaa090 [file file013_nsaa090.docx]

**Supplementary material 3.**

**Extracted coordinates and study details for nociceptive processing meta-analysis (adapted from Tanasescu *et al.,* 2016)**

//Cortical representation of experimental tooth pain in humans

//Jantsch et al 2005; tooth pain weak vs strong; electrical; acute

//

//STUDYID Jantsch 2005 HC Elec

//Subjects=8

-57 -9 22

-55 -9 16

55 -2 14

-35 2 9

-36 -11 15

40 8 4

40 0 7

-3 33 25

-1 2 38

-57 1 18

51 -6 40

48 -9 17

-20 49 1

25 41 2

-38 31 1

54 23 10

37 44 21

-24 56 33

//Cerebral and spinal modulation of pain by emotions SEE WITH CHRIS IF KEEP

//Roy et al 2009; electrical; right stimuli; healthy; pain during unpleasant/pleasant/neutral pictures; below is: Brain activation peaks related to the painful stimulations in all conditions

//STUDYID Roy 2009 HC Elec

//MNI

//Subjects=12

-7 -41 75

7 -48 75

-7 -13 80

7 24 40

-7 -3 40

-3 14 50

-34 17 0

38 14 0

-55 -24 20

69 -24 20

-28 -28 -5

21 -21 -10

41 38 5

-41 45 20

38 42 30

-21 21 -15

13 7 -10

10 0 0

-10 0 0

-7 -10 -15

3 -38 -30

3 -58 -5

3 -83 -25

-31 -52 -45

28 -41 -50

-31 -86 -10

34 -86 0

-31 -61 -10

34 -65 -15

//Dissection of perceptual, motor and autonomic components of brain activity evoked by noxious stimulation

//Piche et al 2010; acute; electrical

//STUDYID Piche 2010 HC Elec

//MNI

//Subjects=11

-3 -45 70

-58 -21 25

65 -24 25

-37 0 10

38 0 5

-3 31 5

7 34 5

-7 14 40

10 17 30

-3 -14 45

7 -7 40

-3 -38 15

10 -38 10

-28 48 30

38 48 30

-24 0 -20

27 -3 -20

-7 -21 0

10 -20 5

-7 -21 -20

-7 -28 -35

7 -28 -25

-34 24 10

34 28 -15

45 38 10

-31 24 30

-28 28 40

41 10 35

-52 3 30

48 3 40

28 -10 50

-55 -38 25

48 -38 50

41 -48 -35

-52 -69 0

52 -58 5

-52 -62 -5

7 -69 5

10 14 0

-10 14 0

0 -58 -25

0 -72 -30

21 -62 30

-21 -62 30

//Emotional and autonomic consequences of spinal cord injury explored using functional brain imaging

//electrical + angry faces; below is healthy controls

//STUDYID Nicotra 2006 HC Elec

//subjects=7

-42 -62 -14

28 -12 -14

2 12 46

-50 -24 54

56 18 -6

//Noxious Somatosensory Stimulation Affects the Default Mode of Brain Function: Evidence from Functional MR Imaging

//Mantini et al 2009; acute; electrical

//STUDYID Mantini 2009 HC Elec

//Subjects=10

-6 -7 43

48 6 19

-33 -31 58

-12 -61 -23

36 -16 13

54 -19 22

-54 2 13

27 -1 19

-12 -16 7

//Right-Lateralized Pain Processing in the Human Cortex: An fMRI Study

//Symonds et al 2006; acute; electrical; right stimuli

//STUDYID Symonds 2006 HC Elec

//Subjects=9

-57 -18 11

42 -5 2

35 14 2

43 33 18

7 18 35

42 5 18

2 16 46

43 -55 41

//Right-Lateralized Pain Processing in the Human Cortex: An fMRI Study

//Symonds et al 2006; acute; electrical; left stimuli

//

//STUDYID Symonds 2006 HC Elec

//Subjects=9

48 -15 14

44 -8 -6

2 -31 27

4 22 29

48 1 10

1 18 49

46 -54 38

//Painful muscle stimulation preferentially activates emotion-related brain regions compared to painful skin stimulation

//Takahashi et al 2011; electrical; skin and muscle; different levels of stimulation and pain intensity; below is Response to skin pain (unrelated to intensity, MI)

//STUDYID Takahashi 2011 HC Elec

//MNI

//subjects=13

34 -22 24

8 -32 30

10 -22 10

18 -46 78

36 -58 -50

-54 0 6

42 52 2

-28 52 16

-8 -28 -32

-20 -60 36

4 -76 52

22 40 -20

46 10 46

34 -56 16

52 -50 56

-54 -44 54

//Anterolateral Prefrontal Cortex Mediates the Analgesic Effect of Expected and Perceived Control over Pain

//Weich et al 2006; electrical; acute; self-controlled vs externally controlled pain; below is : Brain responses to self-controlled AND externally controlled pain

//STUDYID Weich 2006 HC Elec

//MNI

//Subjects=12

57 0 3

45 6 -9

54 18 9

-39 -18 15

-42 3 -9

-54 6 -6

-57 -3 6

-36 0 9

-54 -18 12

-15 6 0

15 6 -3

-15 18 6

12 9 15

12 12 3

-51 -24 27

-63 -27 21

51 -45 33

51 42 -9

24 30 -15

-24 39 -9

-21 21 -6

-57 -42 36

-57 -42 45

-48 -42 27

0 31 2

//Predictability modulates the affective and sensory-discriminative neural processing of pain

//Contrast: High vs Low intensity - Electric HC on right wrist

//STUDYID Carlsson 2006 HC Electric

//subjects=9

-34 24 -12

34 18 -16

-2 52 12

2 38 20

8 16 40

8 6 40

-10 0 42

-10 -16 44

50 26 -6

-50 30 0

36 12 6

-34 10 -4

46 -16 0

-40 -18 -6

42 -22 20

-42 -26 24

-28 -30 64

-42 -26 66

6 -32 -28

10 -24 -10

2 -86 10

-14 -58 -20

16 -46 -18

10 -64 -44

//fMRI Reveals How Pain Modulates Visual Object Processing in the Ventral Visual Stream

//Contrast: Main effect of pain

//STUDYID Bingel 2007 HC Electrical

//subjects=16

36 54 9

-27 51 6

39 -15 18

36 9 9

-30 18 6

15 -12 54

9 36 9

-12 36 9

21 -9 12

27 12 6

-27 15 6

//A simultaneous EEG–fMRI study of painful electric stimulation

//contrast:

//STUDYID Christmann 2006 HC Electrical

//subjects=6

-48 -12 12

-42 -20 52

-14 -25 41

-9 -6 38

-4 -16 51

-2 -53 -12

9 -12 67

17 -48 -20

45 -29 18

45 1 5

-52 -21 18

-43 -5 6

-40 -18 15

-31 17 12

-58 4 26

-50 -17 51

-31 -26 49

-46 -26 54

-33 -23 56

-16 -26 41

-10 -6 36

-4 -20 46

-4 -58 12

8 -14 63

19 -48 -21

49 -29 18

41 10 3

41 5 0

49 -8 9

//Neural correlates of the prolonged salience of painful stimulation

//contrast:Brain regions showing a transient response to the onset and the offset of nonpainful stimulation and a greater tonic response to painful versus nonpainful stimulation

//STUDYID Downar 2003 HC Electrical

//subjects=10

56 -41 21

56 1 18

-49 4 9

8 4 39

-4 10 30

-1 -20 39

14 -17 12

-7 -23 6

-25 -2 -3

47 -26 21

-52 -29 21

41 13 0

-31 19 12

41 -2 3

-37 1 0

-40 -26 48

-1 -35 18

-1 -8 45

11 -50 -21

//Functional topography of the secondary somatosensory cortex for nonpainful and painful stimuli: an fMRI study

//contrast: group analysis of pain stimulation

//STUDYID Ferretti 2003 HC Electrical

//subjects=8

-36 -34 48

-41 -22 18

48 -23 20

-49 -31 19

45 -35 21

-34 -2 15

31 13 15

****************************************************************************************

new update

//Trigeminal activation using chemical, elevtrical, and mechanical sitmuli

//contrast: electrical stim vs baseline

//STUDYID Iannilli 2009 HC Electrical

//subjects=18

45 47 -2

-42 52 -3

42 26 4

45 43 -12

48 36 15

-3 31 40

-42 8 52

42 3 55

39 5 44

53 2 41

53 -42 35

-40 -29 34

-38 -25 46

22 -36 63

54 -28 21

-41 -45 40

50 -59 44

-33 -45 38

-15 -33 29

-12 9 8

9 4 14

47 15 -6

-39 11 -11

-12 -26 12

//Brain processing of pain in patients with unresponsive wakefulness syndrome

//contrast: brain regions activated by pain stimulation in healthy control group (left index finger)

//STUDYID Markl 2013 HC Electrical

//MNI

//subjects=15

-6 17 52

-15 2 1

45 -28 19

51 -31 25

-42 -34 22

-42 -16 7

39 -10 55

51 5 16

//Dissociating Anticipation from perception: acute pain activates default mode network

//contrast: shock max > shock min

//STUDYID Minassian 2012 HC Electrical

//MNI

//subjects=20

-3 0 66

9 -6 12

3 24 33

45 18 -3

9 9 63

63 -42 27

21 -66 42

-18 -18 21

15 -18 -6

57 -27 21

33 -75 24

6 30 18

-18 -18 3

39 -27 42

51 15 24

42 0 48

30 -3 3

3 -21 -15

-12 -6 9

-21 0 6

21 -3 63

3 -33 -6

57 15 0

-12 -24 -12

-6 3 36

33 0 -9

39 -45 42

-18 6 66

36 0 63

-6 -18 -12

-6 -12 -6

21 -66 60

-27 -66 -24

30 -87 -12

36 -63 -21

-12 -57 -18

-3 -78 -24

-36 -54 -45

0 -57 -15

-36 -75 -24

42 -60 -27

-36 -60 -12

3 -69 -39

-39 -84 -15

-33 -96 0

6 -75 -21

18 -66 -48

15 -48 -21

-33 -54 51

-63 -21 30

-57 -39 21

-36 -45 60

-63 -21 21

-15 -66 60

39 39 30

42 42 0

-60 3 3

-57 6 0

-36 24 -9

-36 12 3

-48 -3 0

-30 51 18

-45 -3 48

-51 -66 0

-45 -78 0

9 -27 39

54 -60 0

51 -63 -3

24 -39 -45

6 -87 -6

24 51 -12

9 -54 69

39 51 -15

//Temporal summation of trigeminal pain in human anterior cingulate cortex

//contrast: pain-related hemodynamic responses (fMRI activation)

//STUDYID Obermann 2009 HC Electric

//MNI

//subjects=11

0 10 30

-2 -30 31

42 0 -7

46 -58 57

48 -37 53

8 -16 13

52 22 33

4 16 49

20 4 13

-36 -68 -25

6 -26 -17

//Cortical responses to pain in healthy individuals depends on pain catastrophizing

//contrast: pain related activations for moderate pain

//STUDYID Seminowicz 2006 HC Electric

//subjects=22

32 -29 55

41 -29 25

-53 -19 21

4 3 41

41 -3 20

-28 19 14

14 -19 6

51 0 12

2 -8 67

9 -9 52

12 -22 47

8 -23 -5

//Sex differences in brain activation to anticipated an experienced pain in the medial prefrontal cortex

//contrast: significant activation at intensity 4/5 likert scale stim applied on left

//STUDYID Straube 2009 HC Electric (Female)

//subjects=12

34 11 4

-36 11 10

11 3 41

48 -28 40

-56 -13 43

12 -19 7

-18 -19 4

//Sex differences in brain activation to anticipated an experienced pain in the medial prefrontal cortex

//contrast: significant activation at intensity 4/5 likert scale stim applied on left

//STUDYID Straube 2009 HC Electric (Male)

//subjects=12

41 14 7

-33 20 7

6 7 40

48 -30 37

-49 -13 43

45 -1 10

-45 -7 10

6 -19 1

-12 -19 10

*********************************************************************

//Cortical correlates of perception and suppression of electrically induced pain

//pain-rest

//STUDYID Freund 2007 HC Electric

//MNI

//subjects=15

54 -54 48

-40 -16 16

38 44 -6

40 14 -6

-4 4 18

//Cortical correlates of perception and suppression of electrically induced pain

//pain-rest

//STUDYID Freund 2007 HC Electric

//MNI

//subjects=15

38 52 2

46 0 8

42 -28 10

-54 6 -2

36 6 38

-24 -42 2

//How the pain of others enhances our pain: searching the cerebral correlates of 'compassional hyperalgesia'

//contrast: painful - innocuous

//STUDYID Godinho 2012 HC Electric

//subjects=16

0 -6 51

5 15 35

-5 15 35

-27 -24 72

24 -27 75

-44 -21 21

42 -18 15

42 6 -12

-39 12 9

60 0 12

-60 -30 27

-6 -78 42

-3 -73 42

-33 36 36

36 23 45

6 -20 12

-12 -18 12

//Cerebral somatic pain modulation during autogenic training in fMRI

//contrast: one sample t-test for the condition 'Painful stimulation and resting state'

//STUDYID Naglatzki 2012 HC Electric

//MNI

//subjects=13

6 18 44

60 -24 24

16 -6 66

54 -16 14

60 6 6

-36 10 8

16 -18 10

-10 16 12

//Medial Prefrontal Cortex Activity Is Predictive for Hyperalgesia and Pharmacological Antihyperalgesia

//Seifert et al 2009; pin-prick (mecahnical) hyperalgesia (electrical) and normal pin-prick (mechanical) pain before and after lidocaine; healthy; right stimuli; below is: pin-pick pain before lidocaine

//

//STUDYID Seifert 2009 HC Mech-Pin

//Subjects=12

-54 -22 20

50 -27 19

-41 -26 19

32 9 3

-51 -3 22

52 2 24

//Human brain activity associated with painful mechanical stimulation to muscle and bone

//Maeda et al 2011; mechanical bone and muscle healthy;right stimuli; below is: muscle

//STUDYID Maeda 2011 HC Mech-Press

//MNI

//Subjects=12

-8 12 44

-6 18 32

-12 26 30

28 26 2

34 4 8

-34 6 8

-28 24 -8

62 -32 30

-60 -32 28

-48 -58 42

-44 -48 42

20 -18 10

26 2 6

20 -4 8

-32 6 6

-26 12 10

16 12 8

//Human brain activity associated with painful mechanical stimulation to muscle and bone

//Maeda et al 2011; mechanical bone and muscle healthy;right stimuli; below is: bone

//STUDYID Maeda 2011 HC Mech-Press

//MNI

//Subjects=12

-48 10 6

-48 -52 40

-48 -40 28

-62 -36 32

-30 10 6

//Pain Processing in Medication Overuse Headache: A Functional Magnetic Resonance Imaging (fMRI) Study

//Ferraro et al 2012; mechanical in healthy vs MOH; left stimuli; below is : healthy

//STUDYID Ferraro 2012 HC Mech-Press

//MNI

//Subjects=9

10 36 36

46 16 -8

60 -12 40

-58 -6 8

56 -18 20

56 -36 48

-52 -46 54

62 -28 34

//Secondary somatosensory cortex is important for the sensory-discriminative dimension of pain: a functional MRI study

//Maihofner 2006; acute; mechanical vs thermal; below is MECHANICAL

//

//STUDYID Maihofner 2006 HC

//Subjects=14

-33 -23 52

-40 -10 48

47 -2 45

-30 -61 45

33 -60 45

-52 -23 16

15 -22 18

-49 -26 23

54 -43 39

-48 13 12

48 6 23

-29 25 28

36 37 27

-6 19 55

7 19 53

-46 13 8

38 11 11

-46 13 8

37 -22 15

-2 21 36

3 8 36

//Secondary somatosensory cortex is important for the sensory-discriminative dimension of pain: a functional MRI study

//Maihofner 2006; acute; mechanical vs thermal; below is THERMAL

//STUDYID Maihofner 2006 HC

//Subjects=14

-34 -24 50

-31 -21 49

-30 -60 44

34 -54 45

-42 -21 14

52 -22 14

-46 -26 25

48 -38 45

-40 7 36

44 3 32

-37 42 14

39 33 30

-4 8 60

7 24 51

-28 19 12

35 17 9

-4 10 41

4 8 38

//Brain imaging of mechanically induced muscle versus cutaneous pain

//Uematsu et al 2011; mechanical; skin vs muscle vs both; below is: Region of painful stimulation = SKIN

//STUDYID Uematsu 2011 HC Mech-Press

//MNI

//Subjects=17

52 62 38

54 -30 24

-60 -58 34

-56 -30 22

6 32 52

6 26 36

-8 32 50

-4 26 36

44 14 4

-38 8 8

-34 -20 10

36 16 34

-38 4 38

8 2 -2

-12 -2 2

2 -6 6

-2 -6 6

//Brain imaging of mechanically induced muscle versus cutaneous pain

//Uematsu et al 2011; mechanical; skin vs muscle vs both; below is: Region of painful stimulation = MUSCLE

//STUDYID Uematsu 2011 HC Mech-Press

//MNI

//Subjects=17

46 -66 44

-64 -48 28

2 24 42

2 34 32

-2 38 50

-4 28 36

44 14 6

-30 18 -6

-38 -22 8

40 34 20

-44 38 18

-16 10 -6

4 -12 0

-4 -14 0

//The influence of simultaneous ratings on cortical BOLD effects during painful and non-painful stimulation

//Schoedel et al 2008; acute; mechanical

//Non-rating high intensity pain

//STUDYID Schoedel 2008 HC Mech-IS

//Subjects=11

57 -19 39

46 -37 41

33 21 11

37 -14 16

-30 17 13

-37 -10 14

50 13 9

28 46 33

-17 55 7

38 30 34

43 49 11

48 6 33

48 16 11

-52 11 20

//Representations of Pleasant and Painful Touch in the human orbitofrontal and cingulate cortices

//Rolls et al 2003; mechanical; acute; pain vs pleasant vs neutral;

//STUDYID Rolls 2003 HC Mech-Press

//MNI

//Subjects=8

46 -28 66

-58 -14 8

56 -2 16

36 0 8

-44 -2 10

-60 -32 18

-6 -20 16

10 -22 -18

24 -6 66

22 10 -4

12 -6 4

-26 40 -20

16 32 -24

10 -6 34

8 4 52

//Dynamic assessment of the right lateral frontal cortex response to painful stimulation

//Lopez-Sola et al 2010; mechanical; Stimulus vs No stimulus; acute

//STUDYID Lopez-Sola 2010 HC Mech-Press

//Subjects=25

59 -32 11

-59 -38 15

42 6 42

-38 -5 50

-10 -12 -8

4 -27 -5

38 23 -1

-44 0 4

8 16 49

-18 8 -4

12 10 -2

0 6 35

18 -6 6

-16 -13 6

57 -24 21

-59 -22 25

-20 -73 -28

-56 -25 47

44 41 0

//Touch or pain? Spatio-temporal patterns of cortical fMRI activity following brief mechanical stimuli

//Lui et al 2008; acute; mechanical; noxious vs touch; below are Cortical areas displaying fMRI signal INCREASES following noxious mechanical stimulation

//STUDYID Lui 2008 HC Mech-Press

//Subjects=22

-59 -26 31

-55 -33 38

-51 -23 16

48 -25 49

44 -36 53

59 -19 16

-40 8 -4

-44 -8 0

-55 0 4

4 18 43

0 29 32

-24 11 58

55 12 10

51 17 25

32 6 51

-40 47 9

-44 44 16

40 47 12

48 43 2

48 31 2

/Mapping Brain Response to Pain in Fibromyalgia Patients Using Temporal Analysis of fMRI

//Pujol et al 2009; FM vs healthy (2 different groups of healthy); mechanical; right stimuli; below is : same stimuli producing different pain intensity, HEALTHY GROUP1

//STUDYID Pujol 2009 HC Mech-Press

//Subjects=9

-51 -27 48

-54 -28 26

56 -17 17

-59 -23 15

-36 15 2

36 15 -1

/Mapping Brain Response to Pain in Fibromyalgia Patients Using Temporal Analysis of fMRI

//Pujol et al 2009; FM vs healthy; mechanical; right stimuli; below is : different intensity stimuli producing same pain intensity, HEALTHY GROUP2

//STUDYID Pujol 2009 HC Mech-Press

//Subjects=9

-54 -15 48

-33 -29 62

54 -21 48

-60 -22 26

56 -16 23

-48 -20 18

-33 -2 11

39 -3 -2

-6 -1 36

0 0 55

54 13 35

-30 -62 -17

//Age-related differences in pain sensitivity and regional brain activity evoked by noxious pressure

//contrast: moderately painful versus innocuous

//STUDYID Cole 2010Y HC Mech-press

//MNI

//subjects=15

22 60 18

-36 -62 8

4 32 42

42 48 14

-42 54 12

40 0 58

-40 -4 60

-6 -4 54

-40 -20 68

48 -38 60

-64 -16 42

54 6 4

-46 -20 16

50 -52 54

-52 -50 52

60 -20 18

-68 -38 22

-8 8 34

-2 32 36

44 14 -6

-42 2 6

12 6 16

-10 0 16

22 10 0

-28 4 -6

10 -12 4

-14 -14 4

30 -72 -30

-36 -72 -46

//Age-related differences in pain sensitivity and regional brain activity evoked by noxious pressure

//contrast: moderately painful versus innocuous

//STUDYID Cole 2010 Old HC Mech-press

//MNI

//subjects=15

18 60 20

-32 62 8

0 26 48

48 40 18

-48 42 20

50 6 46

-54 -10 54

-42 -22 68

52 -24 58

-56 -22 54

58 12 2

-44 -16 16

56 -48 46

-56 -50 46

56 -20 12

-68 -16 14

0 32 34

40 18 -4

-40 -6 -2

12 22 8

-18 16 18

-44 -74 -46

**************************************************************************************************

new for new strings and neurosynth

//Pain sensitivity and fMRI pain-related brain activity in Alzheimer's disease

//contrast: Control group main effects of mech pressure in right thumbnail

//STUDYID Cole 2006 HC Mech-press

//MNI

//subjects=15

20 50 30

-18 64 18

54 14 28

-56 22 2

48 14 46

-34 -12 66

-44 -24 62

58 -22 42

-60 -18 48

56 12 4

-60 -26 18

46 -54 52

-64 -34 36

2 26 36

-2 36 30

34 28 0

-42 -2 10

56 10 -2

-62 6 -4

-2 -22 2

-20 28 4

//Activation of central sympathetic networks during innocuous and noxious somatosensory stimulation

//contrast: noxious stimulation

//STUDYID Maihofner 2011 HC Mech-Impact

//subjects=12

33 -24 51

40 -10 48

30 -59 45

-31 -57 44

52 -24 16

-51 -23 17

49 -31 23

-48 -33 24

3 10 36

-2 8 36

46 13 8

-38 12 11

30 14 12

-29 13 11

41 19 29

-40 19 29

46 17 21

-44 16 21

//organisation of felt and seen pain reponses in anterior cingulate cortex

//contrast: main effect of felt pain

//STUDYID Morrison 2007 HC Mech-Press

//subjects=11

-2 7 34

5 27 24

-4 24 30

-33 12 7

39 15 -4

44 -5 8

16 -15 10

-2 52 -16

-31 -77 1

//Brain activity for chronic knee osteoarthritis: dissociating evoked pain from spontaneous pain

//contrast: brain regions identified for painful pressure stimulation of the knee (10 left knee / 4 right knee)

//STUDYID Parks 2011 HC Mech-Press

//MNI

//subjects=9

24 2 60

-48 44 20

36 54 22

-6 10 46

-40 2 -8

40 0 -4

56 16 14

-2 8 52

60 -20 16

-44 20 -10

58 10 -6

-54 -42 22

62 -20 16

-34 -50 56

40 -52 56

-22 -78 36

-24 -2 -12

26 0 -14

-10 10 0

10 8 4

-24 2 -6

28 -4 -6

-16 -22 6

8 -16 10

8 -60 -6

2 -28 -6

//Functional imaging of sensory devline and gain induced by differential noxious stimulation

//contrast: pin-prick stimulation pre-hyperalgesia

//STUDYID Stammler 2008 HC Mech-Press

//subjects=12

-45 -22 51

-33 -51 51

39 -51 47

-51 -27 22

49 -25 21

-40 -53 39

47 -45 29

-41 19 29

40 19 29

14 43 36

-33 18 15

-36 -6 11

38 -5 14

4 -35 20

-12 -17 6

-12 1 19

20 5 5

//Why self-induced pain feels less painful than externally generated pain: distinct brain activation patterns in self- and externally generated pain

//contrast: externally induced pain (left)

//STUDYID Wang 2011 HC Mech-Press

//subjects=8

34 -36 61

-34 -36 61

-39 -28 61

31 -23 61

-6 -17 61

6 -17 61

-5 45 4

5 45 4

-40 -52 52

36 -48 52

-34 15 4

42 10 4

-8 1 0

7 2 0

-10 -44 0

10 -50 0

-3 -69 0

8 -64 0

//Vicarious responses to pain in anterior cingulate cortex: Is empathy a multisensory issue?

//contrast: pain>innocuous stim

//STUDYID Morrison 2004 HC Mech-Press

//subjects=14

62 -16 22

0 -8 58

-58 -24 14

32 -20 58

16 -14 2

//Insular cortex is a traig marker for pain processing in fibromyalgia syndrome - blood oxygenation level-dependent functional magnetic resonance imaging study in Korea

//contrast: high pressure in controls

//STUDYID Kim 2011 HC Mech-Press

//MNI

//subjects=22

-9 30 48

-42 21 48

36 39 -3

-45 12 12

42 42 12

-48 -39 27

54 -27 24

-51 -27 18

51 -21 18

-9 -9 18

9 -9 18

-36 -3 15

42 -21 18

-6 3 12

15 0 21

-51 6 0

48 3 3

39 -9 -18

//Differences in Low Back Pain Behaviour Are Reflected in the Cerebral Response to Tactile Stimulation of the Lower Back

//contrast: intense stimulation of the lumbar region vs. rest (central)

//STUDYID Lloyd 2008 HC Mech-Press

//MNI

//subjects=17

62 -32 38

34 6 4

44 10 -6

54 2 2

62 -20 18

40 -16 -10

62 -18 24

12 -20 -24

4 -34 -30

//Augmented Cerebral Activation by Lumbar Mechanical Stimulus in Chronic Low Back Pain Patients

//contrast: HC Brain activity related to lumbar pressure at VAS 5

//STUDYID Kobayashi 2009 HC Mech-Press

//subjects=8

1 -41 22

1 4 51

30 41 28

36 9 8

//Peltz et al 2011# noxious heat

//stimulus location:Heat, left volar forearm

//STUDYID Peltz 2011 HC Thermal

//TAL

//subjects=11

33 15 5

-32 15 6

36 -8 16

-35 -1 8

50 12 15

-49 11 7

53 -12 18

-50 -5 10

53 -27 47

50 -36 37

-56 -30 28

36 42 23

-30 42 24

7 5 61

-5 2 67

40 5 45

-26 2 61

0 17 35

-2 -29 26

49 -36 35

14 2 7

-19 7 7

//Peltz et al 2011# noxious cold

//stimulus location:Cold, left volar forearm

//STUDYID Peltz 2011 HC Thermal

//TAL

//subjects=11

35 18 8

-32 9 12

34 -15 20

-32 0 17

51 11 8

-46 15 4

51 -8 14

54 -21 26

55 -32 42

-53 -25 25

42 39 15

-46 35 15

43 -3 55

-35 -5 49

7 -11 31

3 18 45

-1 17 43

33 -44 41

//Bornhovd et al 2002#pain intensity

//Stimulus: LEFT hand, painful heat stimuli; infra-red laser

//STUDYID Bornhovd 2002 HC Thermal-H

//MNI305

//subjects=9

54 30 21

-48 27 21

33 6 27

-48 9 21

45 -51 54

-36 -60 57

51 -27 45

-42 -21 54

-6 54 12

-6 36 -12

42 -12 6

-45 -18 12

51 15 -9

-45 12 -9

36 9 0

-42 9 -6

24 0 -24

-27 0 -27

//Brooks et al 2005#Face> Hand+Foot

//stimulus: RIGHT foot, RIGHT hand; thermal resistor

//STUDYID Brooks 2005 HC Thermal-H

//MNI

//subjects=14

-36 24 -6

38 16 2

-38 4 4

40 12 -4

-38 -10 10

-56 -18 14

62 -26 14

-26 4 -8

28 12 -8

44 20 -10

-12 -8 4

16 -10 4

4 -16 4

-14 -16 4

58 -32 20

//Brooks et al 2005#Hand> Face+Foot

//stimulus: RIGHT foot, RIGHT lower lip; thermal resistor

//STUDYID Brooks 2005 HC Thermal-H

//MNI

//subjects=14

-32 22 0

38 16 0

-36 12 0

38 12 2

-38 -16 8

64 -26 14

-22 8 -10

24 12 -6

46 24 -12

12 -8 4

6 -16 -2

-58 -28 14

56 -28 18

//Brooks et al 2005#Foot> Hand+Face

//stimulus: RIGHT foot, lower lip, hand; thermal resistor

//STUDYID Brooks 2005 HC Thermal-H

//MNI

//subjects=14

-34 22 -4

38 14 0

-40 8 -2

-38 -18 8

-56 -10 4

-22 14 -6

26 16 -2

46 22 -12

//Boly et al 2007#Baseline+stimuli perception

//stimulus: LEFT hand; infra-red laser

//STUDYID Boly 2007 HC Thermal-H

//MNI

//subjects=24

42 38 28

-54 6 30

44 42 10

26 -50 38

-44 -36 46

6 24 44

6 -12 2

0 16 32

36 10 -10

-50 8 0

//Boly et al 2007#stimuli perception>baseline

//stimulus: LEFT hand; infra-red laser

//STUDYID Boly 2007 HC Thermal-H

//MNI

//Subjects=24

46 44 12

48 36 26

-50 4 30

18 -68 54

-38 -46 46

6 24 44

6 -54 -32

//Derbyshire et al 2009#T3 constant>T3 offset

//stimulus: thermal, both forearms

//STUDYID Derbyshire 2009B HC Thermal-H

//MNI

//Subjects=12

14 -24 0

-14 16 42

-14 46 4

-38 -54 22

-14 -26 68

36 -20 54

-32 -12 2

42 20 18

//Dube et al 2009#Pain - warm

//stimulus: LEFT leg; thermal

//STUDYID Dube 2009 HC Thermal-H

//MNI

//subjects=12

-56 -36 36

64 -28 24

64 -36 24

-64 -32 28

36 8 12

36 20 -4

-32 28 -8

-40 0 -12

-36 -12 0

0 16 32

4 24 24

0 28 40

-24 0 -16

-16 -8 16

-8 -16 8

-8 -16 -4

8 -12 8

8 20 68

4 16 56

8 24 60

-56 8 12

52 0 52

-52 16 -12

52 16 -12

52 -48 -4

16 8 20

8 16 8

-20 24 8

-20 12 12

32 8 -8

28 -4 8

28 16 -8

-24 20 -4

-24 -76 -48

-44 -52 -36

-28 -72 -28

-32 -56 -52

20 -72 -24

16 -80 -28

28 -68 -28

-28 -40 -48

//Dunckley et al 2005#Midline back > Left foot+Rectum

//10 right handed subjects

//Stimulus: Left foot; thermal resistor

//STUDYID Dunckley 2005 HC Thermal-H

//MNI

//subjects=10

36 20 -6

-36 14 0

38 -20 0

-40 -16 -8

2 20 22

-4 22 24

56 -26 24

-52 -28 18

6 -20 -2

-10 -18 0

14 2 -2

-18 0 -2

8 -16 -10

-6 -20 -12

4 2 48

-4 2 46

60 -40 32

-44 -36 44

-32 40 24

//Dunckley et al 2005#Left foot > Rectum+Midline back

//10 right handed subjects

//Stimulus: Left foot; thermal resistor

//STUDYID Dunckley 2005 HC Thermal-H

//MNI

//subjects=10

42 8 -12

-34 16 4

38 -14 -8

-36 -18 4

10 -6 40

-8 20 26

58 -24 26

-58 -22 22

6 -18 0

-4 -16 -2

12 2 -2

-12 2 -2

2 -30 -8

-10 -14 -8

4 10 52

-8 2 60

50 -32 42

-52 -38 28

//Bingel et al 2002#Left hand + Right hand stimulation

//stimulus: infrared laser to both hands randomly

//STUDYID Bingel 2002 HC Thermal-H

//MNI

//subjects=14

21 -21 -9

18 -6 -18

9 -12 3

39 -54 30

21 9 -6

12 -12 -6

-21 -21 -12

-15 -6 -15

-9 -15 3

39 -57 -27

-27 9 -3

-6 -21 -6

//Strigo et al 2003#Thermal>rest

//stimulus: thermal; upper chest (middle)

//STUDYID Strigo 2003 HC Thermal-H

//MNI

//subjects=7

32 12 8

32 24 6

-36 2 16

-36 12 10

36 -4 -6

-42 0 2

36 -20 14

-28 -22 6

54 -38 32

-56 -24 16

42 50 -2

38 -50 42

-34 -48 38

54 -36 44

-60 -38 38

-2 -26 28

-10 0 42

-16 -14 6

36 -56 -44

28 -56 -30

-40 -52 -44

-26 -66 -32

4 -58 -30

26 6 4

-30 -6 6

//Tseng et al 2010#Noxious heat>rest

//12 healthy right-handed volunteers

//stimulus: RIGHT foot; thermal

//STUDYID Tseng 2010B HC Thermal-H

//MNI

//subjects=12

-64 -26 18

58 -24 22

-36 -20 6

38 -2 6

-14 -44 64

48 2 48

10 -18 -12

-30 34 10

40 54 14

48 18 30

-40 14 -8

-52 8 22

26 10 -20

50 12 22

54 18 18

-34 16 6

36 16 2

-18 -62 -26

36 -52 -38

-14 -6 72

10 16 56

-4 -30 70

-14 -8 6

10 -6 8

-6 2 40

-4 4 42

2 2 44

2 20 32

-30 -18 8

20 10 -10

-6 -18 -14

//Apkarian et al 2000#Large area>small area

//7 right handed subjects

//stimulus: RIGHT hand fingers; hot water thermal

//STUDYID Apkarian 2000B HC Thermal-H

//TAL

//subjects=7

-46 -10 6

-58 -20 28

-50 -22 32

//Becerra et al 2001#Early phase stimulation

//stimulus: LEFT hand*; thermal

//assumption from Becerra 1999

//STUDYID Becerra 2001 HC Thermal-H

//TAL

//subjects=8

3 0 56

15 -6 62

12 9 50

-40 3 12

15 -21 62

37 24 6

-62 6 6

21 33 -6

56 -21 18

53 -30 37

-62 -42 31

34 -6 9

-46 -9 6

-43 -18 18

56 3 6

-62 -15 12

-31 -18 -28

3 3 43

-6 12 25

0 0 34

12 -27 37

18 -6 0

-12 15 3

9 9 9

9 -15 3

-12 -18 9

0 -21 -3

-15 -21 -25

-31 -42 -25

28 -51 -40

-25 -54 -21

//Becerra et al 2001#Late phase stimulation

//stimulus: LEFT hand*; thermal

//assumption from Becerra 1999

//STUDYID Becerra 2001 HC Thermal-H

//TAL

//subjects=8

-15 42 46

12 9 50

-18 -3 40

-6 3 53

53 3 28

25 -12 53

-28 -12 62

-37 33 21

40 39 -6

28 39 21

-46 36 6

43 27 18

-34 24 12

50 15 6

-43 6 12

53 -12 18

28 -30 62

-25 -60 46

53 -24 34

-65 -27 28

53 -36 28

-40 -48 28

9 -42 46

31 18 12

34 6 9

-65 -15 3

-50 -12 -15

-56 -45 3

-65 -48 0

50 -51 6

-46 -54 6

-50 -30 -9

28 -15 -31

-40 -42 -6

-34 -57 -9

-31 -6 -25

9 39 0

0 -9 43

-3 -27 28

-6 -60 12

6 -18 6

-15 -18 9

-18 0 15

-21 -18 -3

3 -21 -21

25 -45 -25

-28 -45 -40

12 -48 -18

-28 -54 -31

-18 -54 -12

//Davis et al 2002#Prickle sensation>deep cold sensation

//stimulus: RIGHT hand; cold evoced prickle

//STUDYID Davis 2002 HC Thermal-C

//TAL

//subjects=7

2 10 36

-40 7 6

38 10 9

-58 -14 15

-40 -44 48

32 -53 48

-52 -26 45

-58 -41 30

56 -26 33

2 -11 54

-43 4 36

-31 -11 54

50 4 24

-19 16 3

2 -20 6

-37 53 30

38 28 33

42 49 12

29 -26 57

//Tracey et al 2000#Heat

//Stimulus: LEFT hand; hot(46c)

//Right handed male subjects

//Separated hot and cold; Laura joined them

//STUDYID Tracey 2000 HC Thermal

//TAL

//subjects=6

37 45 3

40 21 6

53 6 12

56 -12 12

31 -42 59

9 -36 28

-40 18 6

-21 0 -3

6 -18 15

12 -27 3

-3 12 40

-12 12 31

-9 -33 46

//Tracey et al 2000#Heat

//Stimulus: LEFT hand; cold(5c)

//Right handed male subjects

//Separated hot and cold; Laura joined them

//STUDYID Tracey 2000 HC Thermal

//TAL

//subjects=6

28 54 3

46 39 9

0 0 46

46 -12 12

-40 -48 43

-65 -45 21

40 9 0

25 6 15

3 -12 12

12 -30 6

-3 12 40

0 -33 37

//Veldhuijen et al 2009#Prickling

//separated pricking and burning; Laura put them together

//9 right handed

//Stimulus: infrared laser; LEFT foot

//STUDYID Veldhuijen 2009 HC Thermal-H

//TAL

//subjects=10

-8 -1 9

6 -2 7

-37 14 8

-37 -8 16

39 5 19

-2 17 30

36 3 24

-57 -33 25

50 -30 15

-36 -50 -29

//Veldhuijen et al 2009#Burning

//separated pricking and burning; Laura put them together

//9 right handed

//Stimulus: infrared laser; LEFT foot

//STUDYID Veldhuijen 2009 HC Thermal-H

//TAL

//subjects=10

-5 -17 10

11 -3 13

-39 11 3

-30 -5 19

43 17 20

-1 19 37

42 12 31

-56 -39 26

47 -37 20

-38 -53 -37

//Bingel et al 2006#Laser pain>no pain

//right handed subjects

//Stimulus: infrared laser; LEFT and RIGHT hand

//STUDYID Bingel 2006 HC Thermal-H

//MNI

//subjects=19

42 -42 48

57 -21 21

39 -6 0

3 30 39

6 39 9

9 -18 9

18 -3 -15

30 -63 -33

18 12 -3

-54 -42 48

-60 -15 15

-39 6 3

-3 18 48

-6 42 12

-12 -9 0

-33 -72 -21

-15 15 0

//Bingel et al 2007#Laser pain>no pain

//right handed subjects

//3 sessions (day 1,8,22). Results Pooled

//stimulus: 48c heat; LEFT forearm

//STUDYID Bingel 2007 HC Thermal-H

//MNI

//subjects=20

24 -39 69

39 -15 18

39 9 9

9 9 39

9 54 -15

6 -63 36

12 -18 3

24 18 -9

12 -18 -9

30 -63 -24

3 -30 -36

-39 -18 18

-36 6 6

-12 6 36

-6 51 -15

-6 -57 24

-3 -18 3

-15 18 -9

-15 -21 0

-33 -57 -27

-9 -36 -33

//Weich et al 2010#Laser pain>no pain

//right handed

//table s3

//pinprick-like sensation

//Stimulus: laser; RIGHT foot

//STUDYID Weich 2010 HC Thermal-H

//MNI

//subjects=16

3 11 40

-3 -4 43

0 -10 52

-51 11 16

-21 8 -14

-33 23 -23

42 53 16

30 50 37

-42 29 -2

36 50 -17

42 44 -20

36 26 -23

-54 -52 4

-24 44 -14

48 32 34

-3 -16 -20

24 -43 -29

-36 50 -11

9 53 -20

-3 47 16

-30 38 -17

42 -31 -14

51 41 7

-24 -4 1

-39 47 -14

//Remy et al 2003#pain

//Stimulus: thermal (46-49c); LEFT hand

//STUDYID Remy 2003 HC Thermal-H

//MNI

//subjects=12

-30 4 42

-40 16 40

-60 12 26

-14 -74 26

-6 -72 22

-24 -78 30

-6 8 30

//Mobascher et al 2010#pain>no pain; overall activation (Z value)

//stimulus: Laser; LEFT hand

//STUDYID Mobascher 2010a HC Thermal-H

//MNI

//subjects=32

54 -26 20

62 -26 36

38 2 2

52 -20 34

24 0 -20

44 0 58

42 24 -6

-60 -38 22

-36 20 0

-64 -22 22

4 8 36

14 -72 40

-26 -66 -28

10 -16 6

-10 -12 0

8 -18 -18

8 -22 46

//***************ADDED EXTRA STUDIES************************

//**********************************************************

//**********************************************************

//**********************************************************

//**********************************************************

//Baliki et al 2006#Thermal Pain

//Stimulus: thermal; lower back

//STUDYID Baliki 2006 HC Thermal-H

//MNI

//subjects=11

-2 -18 8

4 -20 10

16 12 8

24 8 -2

-24 14 -2

-12 16 8

46 -2 -14

46 -8 -2

-10 -94 -18

-32 -88 -20

0 18 34

2 12 46

18 28 58

-30 18 6

-48 22 -16

-46 4 -2

//Gundel et al 2008#thermal pain pxs only

//right handed women with somatoform pain disorder fulfilling DSM-IV criteria

//stimulus: LEFT forearm

//STUDYID Gundel 2008 HC Thermal-H

//MNI

//Subjects=13

21 -21 15

-12 -9 6

27 15 -3

-27 18 9

9 -18 33

-6 -21 30

39 -18 15

57 -36 24

3 24 51

39 39 0

-45 45 -9

45 -48 51

-57 -48 30

//Ducreux et al 2006# cold pain>no pain

//stimulus: cold; RIGHT hand

//STUDYID Ducreux 2006 HC Thermal-C

//TAL

//Subjects=6

-36 -6 12

40 14 -2

-30 20 -2

50 -46 58

-48 -44 60

8 -78 54

-38 -52 62

2 28 28

-2 30 18

6 28 46

-2 24 44

52 -18 4

-48 -22 10

46 36 12

-44 48 10

30 44 25

20 54 36

4 -8 4

-8 -26 4

14 0 16

-14 -8 18

-16 8 8

-4 -11 53

-43 -18 -10

26 -74 -28

//Maihofner & Handwerker 2005# thermal

//healthy volunteers

//Table 2

//stimulus: Heat; LEFT forearm

//STUDYID Maihofner & Handwerker 2005 HC Thermal-H

//TAL

//subjects=12

29 -34 53

45 -1 39

-42 -44 37

49 -28 26

48 -19 14

-50 -20 13

-32 18 9

39 -16 9

-38 -11 9

-34 40 13

-46 31 4

//Seifert et al 2008#thermal hyperalgesia; main effect only

//stimulus: RIGHT forearm; heat

//STUDYID Seifert 2008 HC Thermal-H

//TAL

//Subjects=14

-35 -32 55

16 -66 43

-25 -54 51

28 50 34

-24 47 32

-3 15 35

//Seifert & Maihofner 2007#Noxious cold

//Induction of experimental cold allodynia with ethonol

//right handed subjects

//stimuls: Cold; RIGHT forearm

//STUDYID Seifert & Maihofner 2007 HC Thermal-C

//TAL

//Subjects=12

-31 13 9

38 19 7

-38 -15 11

-2 26 37

14 15 33

23 -8 -21

6 -10 32

-30 38 13

42 44 12

53 11 26

-52 -34 -5

49 -23 -8

-53 -3 14

-12 9 11

20 11 9

11 -2 7

-22 -66 -6

31 -67 -1

-13 -91 8

//**********************************************************

//**********************************************************

//**********************************************************

// NEW SEARCH

//Valet 2004; Distraction modulates connectivity of the cingulo-frontal cortex

//and the midbrain during pain\97an fMRI analysis

//7 right handed volunteers

//without distraction experiment

//stimulus: RIGHT forearm

//STUDYID Valet 2004 HC Thermal-H

//MNI

//subjects=7

-18 -14 8

12 -14 6

-30 -28 56

-60 -36 22

56 -26 24

-40 -16 14

34 -14 10

-58 -46 36

50 -56 46

-10 -2 46

-10 8 30

-6 -34 24

-36 50 12

36 52 14

-40 6 6

38 16 -4

//Becerra 1999; Human Brain Activation Under Controlled Thermal

//Stimulation and Habituation to Noxious Heat:

//An fMRI Study

//Group 1

//stimulus: heat; LEFT hand

//STUDYID Becerra 1999 HC Thermal-H

//TAL

//subjects=6

25 45 12

-34 15 3

0 12 31

-3 -39 37

9 -21 6

43 -24 40

59 -24 21

-3 -12 58

-37 27 40

-68 -24 3

-59 -42 15

56 -48 3

-59 3 18

-15 -57 -25

//Baliki 2010: PredictingValueofPainandAnalgesia:

//Nucleus Accumbens Response to Noxious Stimuli

//Changes in the Presence of Chronic Pain

//stimulus Thermal: lower back

//Healthy subjects

//STUDYID Baliki 2010 HC Thermal-H

//MNI

//subjects=16

40 38 30

6 -6 62

6 10 38

-38 12 -4

40 8 -2

58 -26 26

-50 -28 26

38 -54 56

-36 -52 56

40 0 56

-38 -8 56

14 -18 6

-16 -18 4

26 4 -2

-22 -4 0

8 -70 -22

-30 -60 22

//Shukla 2011. The analgesic effect of electroacupuncture on

//acute thermal pain perception-a central neural

//correlate study with fMRI

//10 right handed subjects

//stimulus: thermal; LEFT calf

//STUDYID Shukla 2011 HC Thermal-H

//TAL

//subjects=10

47 -47 33

44 1 12

38 13 39

26 49 30

-22 -17 6

5 16 36

//Roberts 2008. Contact heat evoked potentials using simultaneous EEG and fMRI

//and their correlation with evoked pain

//stimulus: heat; LEFT volar fore-arm

//STUDYID Roberts 2008 HC Thermal-H

//TAL

//subjects=10

0 -10 65

24 -16 74

30 -25 68

15 -13 68

12 -13 53

54 -28 38

57 -25 44

45 -25 17

-51 -37 32

-60 -22 29

-39 -4 5

-48 -4 17

-51 -1 32

-60 2 11

24 -40 65

30 -46 56

45 11 5

//------------------------------------------

//new

//Kong 2006 using fMRI to Dissociate Sensory Encoding from....

//Contrast is High>Low pain thermal

//stimulus: right medial aspect of the forearm, thermal

//STUDYID Kong 2006 HC Thermal-H

//MNI305

//Subjects=16

14 -42 72

66 -22 22

-66 -26 20

4 2 46

20 -70 -2

36 -20 16

-36 -16 14

-52 10 -4

-32 0 2

-14 -8 8

16 -14 8

0 -48 -26

-8 -78 18

//Brain Mediators of Predictive Cue Effects on Perceived Pain

//Atlas 2010

//Contrast High Pain Stim> Low Pain Stim

//stimulus heat: left volar forearm

//STUDYID Atlas 2010 HC Thermal-H

//MNI

//Subjects=18

-6 -46 -26

4 -72 -20

40 4 6

-28 30 -8

-46 4 6

-56 -36 26

6 0 44

//Placebo-Induced Changes in fMRI in the Anticipation and Experience of Pain

//Contrast High > Low level pain

//Stimulus heat: left forearm

//STUDYID Wager 2004 HC Thermal-H

//MNI

//Subjects=23

2 40 -6

0 40 8

-6 48 16

0 54 -18

0 -4 50

-54 -8 10

52 -12 14

-38 6 -16

40 -6 -12

-32 4 -28

-60 -38 -8

-6 -2 -18

-40 -22 58

28 -32 64

-24 44 28

22 -102 -6

10 -26 -10

-10 -32 -6

-4 -12 14

0 10 18

-16 -44 -44

-20 -30 -40

26 -68 -40

22 -72 -36

-8 -88 -34

4 -52 -22

//Single trial fMRI reveals signi?cant contralateral bias in responses to laser pain within thalamus and somatosensory cortices

//Contrast - stimulation right hand with Laser

//STUDYID Bingel 2003 HC Thermal-Laser

//MNI

//subjects=14

27 -30 51

-36 -42 51

51 -24 21

-54 -24 24

39 0 9

-39 6 0

9 -3 39

-3 -6 51

12 -9 0

-12 -18 0

//Single trial fMRI reveals signi?cant contralateral bias in responses to laser pain within thalamus and somatosensory cortices

//Contrast - stimulation left-hand with Laser

//STUDYID Bingel 2003 HC Thermal-Laser

//MNI

//subjects=14

27 -30 51

-33 -51 51

54 -27 18

-57 -27 18

36 6 9

-36 0 12

3 -3 42

-9 -6 42

15 -18 9

-12 -15 3

//Regional intensive and temporal patterns of functional MRI activation distinguishing noxious and innocuous contact heat

//contrast: activation due to thermal stimulus on left foot

//STUDYID Moulton 2005 HC Thermal-H

//subjects=33

7 -39 69

-16 -39 66

63 -16 21

-57 -33 18

-64 -25 9

42 22 -3

44 41 9

46 4 23

38 48 2

-50 14 0

-36 36 10

30 19 -5

49 -20 15

1 29 35

0 5 44

3 -20 44

1 -2 48

//Brain Mechanisms Supporting Spatial Discrimination of Pain

//Contrast: Pain main effects / stim - thermal heat on left calf

//STUDYID OSHIRO 2007 HC Thermal-H

//MNI

//subjects=12

32 -44 -44

-26 -74 -50

16 -10 14

-18 -16 8

22 -2 0

-28 -10 2

48 10 -6

-42 2 -4

38 -12 -4

-34 -20 2

6 10 32

-6 6 32

66 -40 28

-68 -30 20

2 2 56

//Brain correlates of subjective reality of physically and psychologically induced pain

//Contrast: activations during laser induced pain (dorsal left hand)

//STUDYID Raij 2005 HC Thermal-laser

//MNI

//subjects=14

38 4 11

-36 5 7

40 -15 14

-37 -14 3

61 -18 21

-59 -20 21

17 -17 15

46 39 0

-34 -65 -24

8 -50 -36

8 4 40

//Memory Traces of Pain in Human Cortex

//Contrast: Stim1 vs rest (47 degrees)

//STUDYID Albanese 2007 HC Thermal-H

//subjects=8

-39 -40 36

51 -31 34

-57 -25 23

52 -22 19

-38 3 10

33 5 10

//Memory Traces of Pain in Human Cortex

//Contrast: Stim1 vs rest (53 degrees)

//STUDYID Albanese 2007 HC Thermal-H

//subjects=8

-40 -39 36

43 -31 40

-54 -25 22

53 -25 21

-42 -1 7

33 -1 10

//Viewing facial expressions of pain engages cortical areas involved in the direct experience of pain

//Contrast: thermal stimulation

//STUDYID Botvinick 2004 HC Thermal-H

//MNI

//subjects=12

6 22 27

-30 -10 28

33 6 11

-24 -3 6

24 4 16

15 7 13

53 -34 24

12 3 58

-30 -74 -29

3 -22 29

42 36 23

//Dissociable Neural Responses Related to Pain Intensity, Stimulus Intensity, and Stimulus Awareness within the Anterior Cingulate Cortex: A Parametric Single-Trial Laser Functional Magnetic Resonance Imaging Study

//CONTRAST: pain-related activity

//STUDYID Buchel 2002 HC Thermal-Laser

//subjects=9

3 6 48

3 6 36

3 9 54

0 18 36

3 27 51

3 24 30

-6 54 12

-6 36 -12

//Neural correlates of interindividual differences in the subjective experience of pain

//contrast: coordinates that show differential activation between high and low sensitivity groups

//STUDYID Coghill 2003 HC Thermal-H

//subjects=17

-2 8 36

-4 18 24

-4 -34 58

30 64 0

32 62 -8

32 52 -18

-18 -20 14

//Brain activity during stimulation of the trigeminal nerve with noxious heat

//contrast: painful stim - warm baseline stim

//STUDYID de Leeuw 2006 HC Thermal-H

//subjects=9

32 37 22

46 6 29

53 -26 25

47 9 1

35 10 8

-34 10 7

1 1 43

4 33 20

-1 33 20

6 -3 9

8 -16 11

-12 -16 11

-35 -24 53

-35 -62 -22

3 -72 36

11 -77 9

//Cerebral activation during hypnotically induced and imagined pain

//contrast: painful stim compared to rest

//STUDYID Derbyshire 2004 HC Thermal-H

//MNI

//subjects=8

-18 -14 10

8 0 4

-6 10 46

8 20 32

14 -72 -16

-58 -28 -12

-38 2 18

-34 12 8

38 14 6

-32 -52 40

30 -70 56

-44 34 34

-48 42 18

-40 54 -2

//Anticipatory brainstem activity predicts neural processing of pain in humans

//contrast: summary of activation during pain period

//STUDYID Fairhurst 2007 HC THermal-H

//MNI

//subjects=12

-4 -32 -8

6 -32 -8

-2 -36 -40

2 -36 -40

-8 -36 -26

6 -36 -22

-4 -20 -18

4 -18 -14

-6 -8 10

8 -8 8

-22 24 -12

42 -6 40

38 -2 4

6 -6 38

44 -18 20

//Distraction Modulates Anterior Cingulate Gyrus Activations during the Cold Pressor Test

//contrast: main effect of cold pressor test

//STUDYID Frankenstein 2001 HC Thermal-C

//MNI

//subjects=10

12 28 22

-8 10 32

0 24 20

52 -26 24

62 -18 20

40 0 18

34 12 12

28 -12 18

-34 50 14

-58 -12 38

//A Comparative fMRI Study of Cortical Representations for Thermal Painful, Vibrotactile, and Motor Performance Tasks

//contrasts: Heat task

//STUDYID Gelnar 1999 HC Thermal-H

//subjects=9

14 -12 72

-6 -22 72

-38 -24 62

-38 -28 62

-44 -26 44

-58 -10 24

-20 -28 36

-42 -10 14

-32 -38 64

************************************************************************************************

New for final search

//Dynamix EEG-Informed fMRI modeling of the pain matrix using 20-ms root mean square segments

//contrast: main effect of laser on dorsum of left hand

//STUDYID Brinkmeyer 2010 HC Thermal-laser

//MNI

//subjects=10

64 -18 40

54 -26 20

40 12 -10

6 22 52

4 18 32

8 -18 48

24 0 -22

-56 -38 24

-34 18 -10

-2 14 24

-4 -62 -44

16 -12 2

-6 -12 12

0 -28 -10

8 -34 -42

//Felt and seen pain evoke the sam elocal patterns of cortical activity in insular and cingulate cortex

//Contrast: noxious versus innocuous

//STUDYID Corradi-Dell'Acqua 2011 HC Thermal-H

//MNI

//subjects=28

36 14 0

26 0 0

34 42 20

0 14 -8

-4 6 32

2 12 24

-30 18 8

-20 2 -2

-62 34 26

-58 20 18

-24 -68 -28

20 -66 -36

62 -38 32

62 -20 22

-34 32 30

50 -28 -12

//Pain-specific modulation of hippocampal activity and functional connectivity during visual encoding

//contrast: pain-related neuronal activation [(Pain + Pic) > (pic only)]

//STUDYID Forkmann 2013 HC Thermal-H

//MNI

//subjects=24

-34 -18 12

-30 16 6

-4 16 44

-18 -60 52

-44 -36 44

-14 -20 8

-38 32 28

38 -16 18

62 -18 22

30 0 54

32 -56 -32

38 -40 42

18 -14 8

16 16 0

//A non-elaborative mental stance and devoupling of executive and pain-related cortices preducts low pain sensitivit in Zen meditators

//contrast: Hot - Warm stimuli in left calf

//STUDYID Grant 2011 HC Thermal-H

//subjects=13

9 23 31

57 -40 37

33 14 -2

-30 20 7

27 47 28

60 11 16

12 -55 19

-33 -34 49

-48 16 40

-30 -58 -41

//Itch Induced by a Novel Method Leads to Limbic Deactivations - A Functional MRI Study

//contrast: brain areas with significant predictor "pain rating"

//STUDYID Herde 2007 HC Thermal-H

//subjects=8

-23 50 31

-13 -5 45

-38 15 15

5 3 44

-53 -21 27

-21 -65 46

-42 -65 2

-32 3 13

-20 11 2

-15 -14 10

-6 -22 10

-5 -51 -14

-24 -46 -23

32 37 29

5 -4 54

25 -17 60

36 -22 56

36 15 15

3 4 34

46 -24 51

39 -35 56

37 -68 2

35 -80 -1

35 -75 -12

17 -5 17

12 -1 2

21 4 8

26 -50 -23

//Cerebral processing of pain in school-aged children with neonatal nociceptive input: an exploratory fMRI study

//contrast: significant brain activations during pain ful heat stimulation for each group (left hand)

//STUDYID Hohmeister 2010 HC Thermal-H

//MNI

//subjects=9

51 -42 57

-30 -51 45

36 18 6

-39 21 3

9 27 63

36 51 -6

45 54 9

54 12 12

-3 33 36

-27 51 27

6 24 54

-6 15 54

27 3 54

//Exploring the brain in pain: Activations, deactivations and their relation

//contrast: signal change evoked by high pain stimuli (right forearm)

//STUDYID Kong 2010 HC Thermal-H

//MNI

//subjects=61

42 18 -8

-40 -16 14

-56 -24 16

6 24 36

-6 20 34

48 48 0

56 -24 22

-30 -32 74

-34 44 28

-2 -40 -48

-34 -62 -32

26 -54 -30

//Neural correlates of symptom formation in functional somatic syndromes: a fMRI study

//contrast: thermal stim versus baseline

//STUDYID Landgrebe 2008 HC THermal-H

//MNI

//subjects=15

36 21 3

-33 15 6

-57 3 42

66 -21 24

48 30 33

48 3 36

6 21 48

42 -48 45

30 6 57

-48 -36 48

//Functional connectivity of the frontoparietal network predicts cognitive modulation of pain

//contrast: LC>HC Low cue>high cue

//STUDYID Kong 2013 HC Thermal-H

//MNI

//subjects=46

-4 36 54

-2 58 20

-20 60 8

-44 38 -10

-34 22 -2

14 64 10

26 60 6

10 46 10

38 16 34

-38 -72 32

-36 -68 50

-52 -66 30

34 -68 30

30 -64 56

8 -80 -34

//Altered anterior insula activation during anticipation and experience of painful stimuli in expert mediators

//contrast: Pain-related regions across both groups

//STUDYID Lutz 2013 HC Thermal-H

//subjects=14

39 7 8

1 3 46

-37 8 8

11 -8 11

9 4 8

52 -29 22

-54 -28 22

39 -21 16

4 -72 -24

31 -31 60

//Laser-evoked potential P2 single-trial amplitudes covary with the fMRI BOLD response int he medial pain system and interconnected subcortical structures

//contrast: brain regions with BOLD activation in response to laser stimulation

//STUDYID Mobascher 2009a HC Thermal-Laser

//MNI

//subjects=20

52 -24 22

60 -44 34

38 22 -2

30 -38 62

26 0 -22

32 -12 62

36 48 18

-64 -20 20

-36 22 0

-64 -24 22

6 12 36

14 -70 38

-14 -46 -34

10 -4 0

-10 -10 4

-6 -14 -20

-6 -20 -28

//Fluctuations in electrodermal activity reveal variations in single trial brain responses to painful laser stimuli - a fMRI/EEG study

//contrast: Brain regions with BOLD activation in response to laser stimulation

//STUDYID Mobascher 2009b HC Thermal-Laser

//MNI

//subjects=12

60 -20 16

58 -42 20

40 4 6

48 -34 60

4 -52 60

24 0 -20

46 44 2

28 -6 70

-62 -22 20

-34 -20 14

-62 -40 28

2 10 40

-34 -52 -36

12 -14 8

-8 -12 0

-8 -20 -16

0 -30 -48

//Neural correlates of perceptual difference between itching and pain: a human fMRI study

//contrast: brain regions activated by pain (left wrist)

//STUDYID Mochizuki 2007 HC Thermal-C

//MNI

//subjects=14

-4 -2 58

36 10 6

-42 10 -4

-8 10 40

-18 6 4

50 -22 12

-2 -4 6

//Painful Heat reveals hyperexcitability of the temporal pole in interictal and ictal migraine states

//contrast: noxious heat activation in the healthy control subjects

//STUDYID Moulton 2011 HC Thermal-H

//MNI

//subjects=11

-34 14 6

36 6 4

44 18 -6

38 18 -14

-38 10 30

-44 14 32

48 14 4

-16 -2 64

2 12 64

-6 -4 66

34 26 48

22 24 40

34 26 40

36 16 56

-26 8 52

-32 0 52

22 10 62

-14 2 52

20 10 50

-22 8 68

48 14 34

12 56 28

-12 24 58

50 16 20

-60 -30 30

58 -34 30

58 -42 24

60 -16 30

-54 -36 44

-52 4 -2

42 8 -34

16 -24 42

40 -32 22

-10 2 12

-10 -2 14

-16 0 18

-8 4 8

16 -78 -48

-6 -24 -28

12 -62 -54

-6 -60 -38

-4 -24 -22

-10 -22 -24

-14 -74 -36

0 -34 -8

//BOLD responses in somatosensory cortices better reglect heat sensation than pain

//contrast: high-painful heat activation detected by the three EV model (early interval and late) in right cheek

//STUDYID Moulton 2012 HC Thermal-H

//MNI

//subjects=12

42 18 -10

-32 14 2

38 52 10

46 -48 50

4 28 34

48 18 -6

52 -34 40

40 40 2

14 -4 14

48 -44 54

30 52 22

//Neural correlates of individual differences in pain-related fear and anxiety

//contrast: group activations for pain > Warmth

//STUDYID Ochsner 2006 HC Thermal-H

//MNI

//subjects=13

0 -12 68

44 32 24

54 4 52

48 8 36

38 42 2

6 8 36

-2 -28 34

28 16 0

42 0 10

38 -22 12

44 -32 26

-70 2 2

54 -38 12

54 -36 32

66 -46 40

-42 -68 40

-20 0 14

-8 -2 6

16 -6 14

//Brain Mechanisms supporting discrimination of sensory features of pain: a new model

//contrast: pain-related activations

//STUDYID Oshiro 2009 HC Thermal-H

//subjects=12

28 -52 -30

-22 -64 -24

18 -14 14

-14 -8 14

22 -2 -2

-24 12 -2

32 10 2

-36 6 -2

34 -14 4

-34 -24 -2

48 4 28

-28 56 16

8 4 38

-4 12 34

48 -50 44

-48 -36 44

58 -32 32

-56 -34 22

6 -4 60

-6 -6 66

//The 'where' and the 'when' of the BOLD response to pain in the insular cortex. Discussion on amplitudes and latencies.

//contrast: painful stimulation

//STUDYID Pomares 2013 HC Thermal-Laser

//MNI

//subjects=21

-36 14 -4

-28 16 0

38 18 -4

-2 28 28

-8 16 34

2 22 34

-38 -2 14

-38 -14 16

40 -14 16

40 0 8

-30 44 18

-36 46 10

-34 32 22

40 38 12

38 40 4

-54 6 12

-30 -56 -30

-22 -60 -34

-10 -74 -42

-8 -74 -24

-14 -66 -28

34 -54 -28

16 -54 -20

26 -52 -24

24 -70 -18

-58 -18 18

58 -14 18

-46 -50 4

58 -42 10

-18 -38 0

0 -52 -30

6 -64 -14

44 -24 -6

-4 -12 0

//Brain processing of the signals ascending through unmyelinated C fibers in humans: an event-related functional magnetic resonance imaging study

//contrast: coordinates of regions activated by both c and A nociceptor stimulation

//STUDYID Qui 2006 HC THermal-Laser

//MNI

//subjects=13

8 -6 12

-12 -4 14

62 -20 18

-64 -26 18

58 4 4

-6 4 42

2 10 48

6 22 50

30 21 3

-31 25 1

//Pain processing in patients with migraine: an event-related fMRI study during trigeminal nociceptive stimulation

//contrast: stimulus reltaed functional changed at 53 degrees Celsius in patients

//STUDYID Russo 2012 HC Thermal-H

//subjects=16

53 -5 42

-46 -14 45

29 37 27

-28 37 33

38 28 9

-34 25 6

2 4 42

53 -23 12

53 35 27

-61 -26 21

35 -20 12

11 -23 6

44 -38 9

-25 -62 43

//Brain activity during sympathetic response in anticipation and experience of pain

//contrast: pain experience (left forearm)

//STUDYID Seifert 2012 HC Thermal-H

//subjects=9

-58 -36 26

51 -25 27

-35 11 9

38 11 14

-42 -5 11

37 -20 24

-4 11 40

-36 39 31

29 43 34

42 36 11

5 8 43

-51 10 13

46 10 18

-36 28 4

-14 -3 60

-20 3 15

25 15 2

-17 -9 20

-4 -22 13

6 -22 17

-31 -61 -23

-5 -61 -15

35 -54 -23

3 -63 -14

28 -73 -19

-24 -54 32

-33 -51 7

34 -44 27

//Mapping pain activation and connectivity of the human habenula

//contrast: early and late phase overlap positive activation

//STUDYID Shelton 2012 HC Thermal-H

//MNI

//subjects=11

-30 -28 6

-28 42 24

-38 42 26

-48 22 -4

-42 16 46

-54 14 20

-48 10 22

-46 8 40

40 4 34

-48 2 34

-60 -40 40

-44 -42 56

-50 12 -8

-62 -38 18

14 -86 -14

20 -90 -12

-40 10 0

-44 -6 -4

-18 4 0

12 -8 8

8 -18 8

-6 -18 8

18 -20 10

12 -22 4

4 -23 4

-4 -24 4

40 -48 -56

28 -64 -34

6 -78 -28

4 -82 -26

//Functional MRI brain imaging studies using the contact heat evoked potential stimulator (CHEPS) in a human volunteer topical capsaicin pain model

//contrast: summary of BOLD activation following CHEPS precapsaicin (left)

//STUDYID Shenoy 2011 HC THermal-H

//subjects=12

54 -29 48

36 13 6

9 13 45

45 -5 60

51 10 15

6 28 27

27 -59 -21

-24 -47 63

-45 4 6

-9 -23 39

-38 37 30

-57 10 9

-27 -62 -18

-15 -20 12

//Roles of the insular cortex in the modulation of pain: insights from brain lesions

//contrast: pain-related activation from stimulated left calf

//STUDYID Starr 2009 HC Thermal-H

//subjects=13

6 -84 -34

-30 -70 -26

4 -24 4

16 10 0

-30 0 2

38 8 8

-36 4 6

38 -18 12

4 16 30

-4 8 38

54 -38 42

4 -34 74

66 -34 22

-66 -22 16

8 -16 72

-6 -2 64

//Roles of the insular cortex in the modulation of pain: insights from brain lesions

//contrast: pain-related activation from stimulated right calf

//STUDYID Starr 2009 HC Thermal-H

//subjects=13

20 -88 -34

-26 -76 -30

10 -4 4

-12 14 -4

18 -2 -8

-18 -20 12

36 26 0

-34 12 -2

-32 -18 8

6 10 34

-6 2 38

54 -40 28

-22 -42 62

-10 -24 72

62 -22 16

-56 6 6

0 2 50

-10 -14 64

//fMRI evidence of degeneration-induced neuropathic pain in diabetes: enhanced limbic and striatal activations

//contrast: group activation areas in the control group (stim right)

//STUDYID Tseng 2012 HC Thermal-H

//MNI

//subjects=11

44 -10 14

-6 12 30

2 -10 46

-44 10 -12

42 14 -2

12 -4 6

4 -58 -28

//Changes in brain function and morphology in patients with recurring herpes simplex virus infections and chronic pain

//contrast: Painful > warm stimulated both sides

//STUDYID Vertiainen 2009 HC Thermal-H

//subjects=11

1 -13 32

5 14 33

56 -42 44

-60 -41 37

41 1 36

50 -27 26

43 49 19

45 -11 12

-37 -18 10

2 -18 3

-15 6 0

16 6 0

43 7 8

-37 5 7

-41 -65 -34

*************************************************************************************************

Last additions

//Functional magnetic resonance imaging of capsaicin induced thermal hyperalgesia

//contrast: pre-capsaicin stim right

//STUDYID Brooks 2003 Thermal HC

//subjects=16

36 18 7

-33 6 -1

3 -28 29

3 17 52

-45 -1 30

51 8 47

36 -45 46

-27 -60 -25

36 -68 -19

0 -3 8

33 48 28

//Altered Pain Processing in Veterans With Posttraumatic Stress Disorder

//contrast: individual temerature

//STUDYID Geuze 2007 Thermal HC

//subjects=12

8 20 43

-2 20 42

36 -53 35

-44 -47 36

42 14 34

29 46 0

-30 27 28

42 43 3

49 13 3

53 -3 5

-49 -2 6

-40 15 1

38 15 1

2 -15 -1

-17 4 4

//FMRI reveals abnormal central processing of sensory and pain stimuli in ill Gulf War veterans

//contrast: noxious stim

//STUDYID Gopinath 2012 Thermal HC

//subjects=14

55 16 28

43 14 16

35 -17 -6

24 59 30

4 22 62

27 14 53

4 -14 29

35 39 51

47 -7 -7

19 8 -15

23 13 -22

8 20 4

16 17 4

17 -20 -1

19 -8 -7

11 46 -16

4 -46 23

-54 20 35

-52 22 23

-32 -25 2

-5 65 51

-9 8 59

-19 11 57

-11 4 35

-62 38 28

-40 -21 -30

-28 31 -15

-7 23 8

-18 24 4

-19 -17 8

-20 -15 1

-25 59 -24

-3 31 -32

//The subjective experience of pain: Where expectations become reality

//contrast: pain-induced signal changes stim right

//STUDYID Koyama 2005 Thermal HC

//subjects=10

2 -50 -24

24 -42 -54

-26 -72 -30

14 -18 4

-12 -10 16

26 -2 8

-30 0 4

40 26 6

-38 4 0

44 -12 -4

-36 -20 14

30 44 24

8 16 38

-2 -4 44

-54 -38 32

-4 -18 66

-18 -38 64

//Early Decay of Pain-related Cerebral Activation in Functional Magnetic Resonance Imaging

//contrast:Right arm stimulation

//STUDYID Kurata 2002 THermal HC Right

//subjects=5

62 -30 27

51 -45 34

-61 -29 16

-55 -34 38

-55 0 5

-63 -15 9

48 11 1

1 -1 43

47 -1 30

39 -12 53

-57 0 26

-39 5 10

-43 -11 13

42 41 21

-27 26 36

44 34 8

22 3 12

19 6 2

//The Cross-Modal Interaction Between Pain-Related and Saccade-Related Cerebral Activation: A Preliminary Study by Event-Related Functional Magnetic Resonance Imaging

//contrast: pain activation right stim

//STUDYID Kurata 2005 Thermal HC

//subjects=6

53 -23 24

-57 -14 21

//Effects of Duloxetine Treatment on Brain Response to Painful Stimulation in Major Depressive Disorder

//contrast: main effect of painful heat

//STUDYID Lopez-Sola 2010 Thermal HC

//MNI

//subjects=20

-36 0 -8

40 34 2

-36 32 12

46 34 36

-30 -8 -6

30 6 6

6 20 36

4 -50 -18

//A Pilot Functional MRI Study of the Effects of Prefrontal rTMS on Pain Perception

//contrast: areas of activation associated with the pain minus rest contrast in all 23 subjects

//STUDYID Martin 2013 Thermal HC

//subjects=23

27.41 23.26 6.75

36.81 6.11 5.7

25.44 4.2 7.31

50.03 0.19 7.2

0.85 22.21 26.43

-31.28 21.76 8.1

//The val158met polymorphism of human catechol-O-methyltransferase (COMT) affects anterior cingulate cortex activation in response to painful laser stimulation

//contrast: activation in response to alser stimulation (left)

//STUDYID Mobascher 2010b Thermal HC

//MNI

//subjects=57

62 -24 20

36 24 -4

42 -36 58

22 0 -16

44 -2 58

46 38 2

-58 -24 18

-36 20 2

-38 44 20

-24 0 -18

6 12 34

12 -70 40

-30 -60 -34

14 -10 2

12 -12 -2

10 -24 -14

2 -30 -46

//Functional magnetic resonance imaging response to experimental pain in drug-free patients with schizophrenia

//contrast: pain tolerance vs. non-painful stimulus in controls

//STUDYID de la Fuente-Sandoval 2010 Thermal HC

//MNI

//subjects=13

18 -15 -12

45 -9 -6

6 0 -9

-12 12 30

-6 -30 27

-51 -48 54

21 -75 57

-36 -57 66

//Central representation of cold-evoked pain relief in capsaicin induced pain: An event-related fMRI study

//contrast: 43 degrees main effect

//STUDYID Mohr 2009 Thermal HC

//MNI

//subjects=15

6 26 44

2 16 54

-8 26 26

-42 28 32

-40 42 28

26 42 16

40 52 16

-60 -22 22

62 -24 28

56 -42 34

-32 16 8

42 6 -4

36 -22 -6

-12 -12 -6

14 -22 4

-34 2 -16

30 8 -22

12 -28 -26

14 -36 -34

-6 -60 -4

26 -44 -36

-40 -50 -38

//Central representation of cold-evoked pain relief in capsaicin induced pain: An event-related fMRI study

//contrast: 0 degrees main effect

//STUDYID Mohr 2009 Thermal HC

//MNI

//subjects=15

12 16 60

-8 22 24

14 32 24

54 34 14

48 46 18

-66 -20 22

54 -42 32

-52 -38 54

-52 2 4

42 -6 2

-52 -18 12

32 -6 10

-10 -16 2

-6 -6 2

38 8 -18

16 -36 -36

-6 -66 -30

-44 -50 -38

28 -54 -22

//Prestimulus functional connectivity determines pain perception in humans

//contrast: pain>no pain

//STUDYID Ploner 2010 Thermal HC

//MNI

//subjects=16

-48 4 -2

30 22 10

-44 -4 10

34 -16 8

-52 -24 20

50 2 6

-46 -36 42

50 -32 26

-2 10 34

-2 -12 68

-38 32 23

32 46 12

-12 -14 10

14 -6 8

-30 -2 0

20 6 -4

4 -22 -20

//Cerebellar responses during anticipation of noxious stimuli in subjects recovered from depression

//contrast: noxious vs non-noxious

//STUDYID Smith 2002 Thermal HC

//MNI

//subjects=8

34 22 -2

-30 20 -2

58 8 -2

-52 -10 6

2 12 34

-56 -20 16

58 -18 14

40 42 18

-36 42 18

2 -72 2

28 -62 -32

-30 -76 -30

-38 -56 -30

//The stress model of chronic pain: evidence from basal cortisol and hippocampal structure and function in humans

//contrast: pain vs warm

//STUDYID Vachon-Presseau 2013 Thermal HC

//MNI

//subjects=18

-6 12 36

4 -2 44

-40 8 0

36 6 14

54 -24 22

-54 -30 28

58 4 6

-56 -2 4

10 -2 74

16 -4 -4

-16 -14 -4

2 -28 -12

-10 -36 66

24 -42 68

-2 -54 -28

-28 -64 -18

-16 -66 14

//Pain and non-pain processing during hypnosis: A thulium-YAG event-related fMRI study

//contrast: pain activation in normal wakefulness

//STUDYID Vanhaudenhuyse 2009 Thermal HC

//MNI

//subjects=13

0 -22 -14

6 -18 16

-14 10 8

16 6 -2

-60 -34 10

40 -14 14

48 -34 52

-6 18 44

34 48 12

36 14 60

//Individual Sensitivity to Pain Expectancy is Related to Differential Activation of the Hippocampus and Amygdala

//contrast: pain vs. baseline (innocuous)

//STUDYID Ziv 2010 Thermal HC

//subjects=10

53 -36 36

-57 -27 27

52 1 44

-36 -8 40

-5 3 48

5 3 51

3 2 36

-5 5 45

38 13 11

-36 17 16

23 6 8

-23 11 5

13 -10 11

-11 -11 7

-64 -11 8

//The Effect of Treatment Expectation on Drug Efficacy: Imaging the Analgesic Benefit of the Opioid Remifentanil

//contrast: effects of painful thermal stimulation

//STUDYID Bingel 2011 Thermal HC

//MNI

//subjects=22

16 -44 70

52 -26 28

38 42 21

22 44 -14

6 10 42

36 6 8

12 10 2

12 -8 2

36 -68 -2

10 -22 -16

-12 -44 70

-60 -22 24

-34 36 24

-22 38 -14

6 14 36

-34 4 1

-12 8 0

-16 -12 8

-26 -68 -34

-6 -24 -16

//Treating pain with pain: Supraspinal mechanisms of endogenous analgesia elicited by heterotopic noxious conditioning stimulation

//contrast: phasic thermal pain during saline

//STUDYID Sprenger 2011 Thermal HC

//MNI

//subjects=22

-46 -26 48

-50 -24 48

44 -30 42

48 -32 44

34 -42 40

-56 -18 24

62 -16 24

60 -16 30

-38 -20 54

-32 16 4

-38 16 -2

-46 2 4

34 20 4

-32 44 28

42 42 14

48 24 28

-4 12 48

-4 20 38

6 20 36

-2 -20 30

4 -26 26

-14 -18 4

12 -14 6

-24 6 -4

20 12 -2

-6 -26 -6

-6 -18 -4

6 -26 -4

2 -20 -22

2 -38 -38

-38 -56 -50

-20 -72 -48

24 -52 -22

22 -58 -50

34 -50 -48

//Functional Imaging of Pain in Patients with Primary Fibromyalgia

//contrast: pain vs. baseline (stimulus left)

//STUDYID Cook 2004 HC Thermal-H

//subjects=9

40 16 -2

62 -10 6

32 42 34

-38 -22 10

-20 -24 42

-24 56 24

//Perception and Suppression of thermally induced pain: a fMRI study

//contrast: pain-rest

//STUDYID Freund 2009 Thermal HC

//MNI

//subjects=15

44 24 -4

50 24 28

42 -62 54

12 24 48

56 -36 44

//Perception and Suppression of thermally induced pain: a fMRI study

//contrast: pain-rest

//STUDYID Freund 2009 Thermal HC

//MNI

//subjects=15

26 10 -8

-34 26 0

12 24 46

-12 -80 -36

22 -12 68

//Sweet-taste induced analgesia: an fMRI study

//contrast: nonsweet contrast vs. baseline

//STUDYID Kakeda 2010 Thermal-C HC

//MNI

//subjects=12

4 14 44

30 12 6

22 2 2

-36 6 2

-22 6 2

-58 6 18

-20 -74 -28

54 -44 40

-60 -50 36

40 46 24

40 28 44

//fMRI of Thermal Pain: Effects of Stimulus Laterality and Attention

//contrast: RA (paying attention to thermal stimulus on right hand)

//STUDYID Brooks 2002 Thermal-H HC

//subjects=18

36 18 5

-30 24 7

-39 -20 20

66 -16 23

-63 -22 23

57 8 -1

-57 -3 0

6 16 32

-9 11 35

0 -19 29

24 -60 -27

-36 -60 -33

//fMRI of Thermal Pain: Effects of Stimulus Laterality and Attention

//contrast: RA (paying attention to thermal stimulus on left hand)

//STUDYID Brooks 2002 Thermal-H HC

//subjects=17

42 20 -6

-36 23 -4

-36 12 2

42 -17 20

57 9 2

9 19 38

-3 19 35

-18 -69 -20

-54 -6 9

39 42 26

-27 36 20

//Cerebral activation during thermal stimulation of patients who have burning mouth disorder:an fMRI study

//contrast: painful vs. baseline (innocuous)

//STUDYID Albuquerque 2006 Thermal-H HC

//subjects=8

14.4 -58.1 -26.8

4.8 53.1 -0.4

-26.2 -50.1 -32.1

-12.9 -17.5 13.6

28.3 3.4 12.4

0.2 37.7 30.2

9.7 -13.5 12.4

40 3.1 32

33.1 30.7 29.8

50.2 -41.9 31.5

//UPDATE to 2015

//STUDYID Gard 2011 HC Elec A

//subjects=17

42 2 -8

57 -7 25

39 8 31

15 -16 1

15 14 4

3 2 61

0 -16 37

-36 -7 1

-24 44 25

-42 17 40

-45 -1 31

-54 -22 31

//STUDYID Gard 2011 HC Elec B

//subjects=17

33 -19 7

51 -28 -8

39 -1 46

39 -46 37

36 -31 58

18 -46 55

9 19 1

12 2 1

9 -28 55

6 8 37

0 -28 28

-15 -1 1

-39 -4 -5

-48 -34 34

-45 -46 40

//STUDYID Ibinson 2013 HC Elec

//MNI

//subjects=15

-56 -22 44

58 -18 18

-52 -22 14

-2 -12 44

0 26 28

44 -4 6

-38 -4 10

-16 -24 -4

4 18 58

18 -54 -24

//STUDYID Ibinson 2013 HC Elec

//MNI

//subjects=15

-46 -40 58

56 -26 20

-64 -22 20

0 -32 22

56 16 -10

-50 -4 0

52 -50 40

22 -52 -26

-40 -58 -30

//STUDYID Kim 2013 HC Mech

//MNI

//subjects=11

50 -3 26

28 58 8

30 -70 -20

46 36 8

40 48 16

16 -12 12

-60 -32 32

-36 -62 -26

-2 -72 10

//STUDYID Kamping 2013 HC Thermal

//MNI

//subjects=16

-15 -84 -36

-12 -78 46

57 -51 42

54 -36 36

-60 -27 21

-9 21 48

-36 -9 60

33 -3 63

-30 18 0

42 24 12

-36 33 33

-9 -57 18

-33 -39 -9

33 -36 -12

-6 51 -9

//STUDYID Kucyi 2013 HC elec

//MNI

//subjects=51

36 14 -6

-30 18 4

-38 14 -6

36 2 4

42 -18 12

6 22 32

-4 -20 26

54 -44 38

-32 42 16

42 40 20

-44 20 32

26 46 18

36 6 34

-30 -58 40

14 -68 34

-8 -72 36

42 -22 44

56 -36 -14

-54 -36 -12

10 -88 -4

18 -26 -8

-40 -60 -34

//STUDYID Perini 2013 HC Therm

//subjects=18

42 46 18

-37 37 12

35 13 6

-34 7 12

38 20 18

56 7 5

-49 7 9

//STUDYID La Cesa 2014 HC Therm

//MNI

//subjects=12

52 -2 -30

26 -24 -16

28 -42 -14

20 34 38

-38 20 52

40 10 -36

-18 34 44

-44 6 -40

-20 44 18

-34 -26 -14

-54 -2 -32

-48 6 -22

-40 22 24

40 -36 6

26 38 44

-36 -26 -8

-8 40 -4

-36 -22 -20

-18 -32 -16

24 0 -14

18 -34 10

-14 -34 8

-38 8 -10

2 -34 -10

//STUDYID Misra 2014 HC Therm

//MNI

//subjects=15

0 26 31

-1 0 61

0 12 57

-47 -13 13

-26 -5 8

-22 8 -6

-16 17 6

-60 -33 24

-39 -40 47

-28 -52 51

-21 -74 -50

-30 -91 -1

-45 -70 3

39 16 3

24 10 -5

11 8 8

13 17 0

62 -28 20

50 -35 46

33 -60 -23

31 -87 -2

43 -76 -10

28 -68 35

40 4 -13

55 5 31

//STUDYID Nickel 2014 HC Elec

//subjects=48

51 -22 22

-51 -22 19

0 20 34

0 20 34

36 14 4

-39 -4 10

51 -1 13

-51 -1 10

-1 -10 10

-12 -16 7

0 -31 25

0 -31 25

12 2 7

-29 -3 13

48 -43 40

-54 -40 37

-48 -55 4

42 41 16

-36 35 19

3 17 46

-3 -4 58

9 -22 -8

-9 -19 -5

24 -52 -23

-24 -55 -23

//STUDYID Sofina 2014 HC Therm

//MNI

//subjects=4

56 6 -2

-60 -52 26

44 -28 34

16 -12 16

66 -44 28

68 -14 18

68 -36 26

-38 16 -4

//STUDYID Sofina 2014 HC Therm

//MNI

//subjects=4

36 56 20

68 -48 4

48 -36 62

0 16 40

34 -12 66

42 -18 64

-50 12 34

40 8 4

//STUDYID Theysohn 2014 HC Elec

//MNI

//subjects=14

20 -44 76

50 -18 16

-46 -50 46

34 10 12

-38 -14 -4

32 -26 22

-6 -4 32

30 44 -6

48 -18 18

20 -88 -24

//STUDYID Wey 2014 HC Mech

//MNI

//subjects=11

-42 -6 6

34 -42 4

16 32 -18

-54 -76 16

-34 -20 32

-24 -88 -30

20 32 8

10 10 4

-40 -36 14

//STUDYID Bogdanov 2015 HC Thermal

//MNI

//subjects=24

-3 -73 49

-57 -70 -75

45 5 58

27 -4 -8

6 -40 46

3 -43 16

12 47 -11

-9 29 -17

48 23 28

//STUDYID Bogdanov 2015 HC Thermal

//MNI

//subjects=24

-21 -25 25

18 -16 28

-18 -1 25

-18 -13 28

12 -37 13

-33 -22 19

-21 -25 25

-12 -19 19

//STUDYID Bogdanov 2015 HC Thermal

//MNI

//subjects=24

-12 29 -17

3 35 -17

//STUDYID Bogdanov 2015 HC Thermal

//MNI

//subjects=24

36 17 -2

-30 20 -5

45 -16 16

-39 -7 -2

-30 14 7

42 -7 10

48 11 19

18 -4 -14

-21 -4 -14

-15 8 -11

9 2 4

3 -22 31

-60 -28 22

63 -22 22

0 20 25

-60 -49 43

33 -49 43

60 -40 37

48 38 7

-15 -34 -8

-18 -7 -14

6 -28 4

12 -10 10

54 -10 16

27 29 -17

12 -64 31

-48 38 19

36 53 31

6 11 61

0 -31 -5

-60 -58 7

-18 -73 -32

12 -91 -26

-9 8 4

9 5 7
